# Signatures of host specialization and a recent transposable element burst in the dynamic one-speed genome of the fungal barley powdery mildew pathogen

**DOI:** 10.1186/s12864-018-4750-6

**Published:** 2018-05-22

**Authors:** Lamprinos Frantzeskakis, Barbara Kracher, Stefan Kusch, Makoto Yoshikawa-Maekawa, Saskia Bauer, Carsten Pedersen, Pietro D. Spanu, Takaki Maekawa, Paul Schulze-Lefert, Ralph Panstruga

**Affiliations:** 1 Institute for Biology I, Unit of Plant Molecular Cell Biology, RWTH Aachen University, Worringerweg 1, 52056 Aachen, Germany; 2 Max Planck Institute for Plant Breeding Research, Department of Plant-Microbe Interactions, Carl-von-Linné-Weg 10, 50829 Cologne, Germany; 3 Department of Plant and Environmental Sciences, University of Copenhagen, Thorvaldsensvej 40, 1871 Frederiksberg, Denmark; 4 Imperial College, Department of Life Sciences, Sir Alexander Fleming Building, London, SW7 2AZ UK

**Keywords:** Co-evolution, Copy number variation, Effectorome, Evolutionary genomics, Fungal genomics, Host specialization, Synteny, Transposable elements

## Abstract

**Background:**

Powdery mildews are biotrophic pathogenic fungi infecting a number of economically important plants. The grass powdery mildew, *Blumeria graminis*, has become a model organism to study host specialization of obligate biotrophic fungal pathogens. We resolved the large-scale genomic architecture of *B. graminis forma specialis hordei* (*Bgh*) to explore the potential influence of its genome organization on the co-evolutionary process with its host plant, barley (*Hordeum vulgare*).

**Results:**

The near-chromosome level assemblies of the *Bgh* reference isolate DH14 and one of the most diversified isolates, RACE1, enabled a comparative analysis of these haploid genomes, which are highly enriched with transposable elements (TEs). We found largely retained genome synteny and gene repertoires, yet detected copy number variation (CNV) of secretion signal peptide-containing protein-coding genes (*SPs*) and locally disrupted synteny blocks. Genes coding for sequence-related SPs are often locally clustered, but neither the *SPs* nor the TEs reside preferentially in genomic regions with unique features. Extended comparative analysis with different host-specific *B. graminis formae speciales* revealed the existence of a core suite of *SPs*, but also isolate-specific *SP* sets as well as congruence of *SP* CNV and phylogenetic relationship. We further detected evidence for a recent, lineage-specific expansion of TEs in the *Bgh* genome.

**Conclusions:**

The characteristics of the *Bgh* genome (largely retained synteny, CNV of *SP* genes, recently proliferated TEs and a lack of significant compartmentalization) are consistent with a “one-speed” genome that differs in its architecture and (co-)evolutionary pattern from the “two-speed” genomes reported for several other filamentous phytopathogens.

**Electronic supplementary material:**

The online version of this article (10.1186/s12864-018-4750-6) contains supplementary material, which is available to authorized users.

## Background

Powdery mildews (Ascomycota, Erysiphales) are ubiquitous fungal plant pathogens in temperate regions of the world [1]. They thrive on the basis of an obligate biotrophic lifestyle, i.e., by retrieving nutrients from living plant cells for fungal growth and reproduction, and have been extensively studied regarding molecular and genetic interactions with both host [2] and non-host plants [3]. Despite advances in the deployment of durable resistance [4], powdery mildews remain a constant threat for economically important crops as they rapidly evade selection pressure resulting from fungicide application [5, 6] and resistance (*R*)-gene mediated immunity [7]. The barley powdery mildew pathogen, *Blumeria graminis* f.sp. *hordei* (*Bgh*), is a member of the species *Blumeria graminis* that is specialized on its host plant, barley (*Hordeum vulgare*). There are various specialized forms (*formae speciales*) of *B. graminis*, where each *forma specialis* (f.sp.) is capable of infecting the respective host plant species belonging to the grasses (Poaceae) family, including cereals [8]. Within each *forma specialis*, numerous isolates (strains) can be differentiated, primarily based on their respective virulence/avirulence phenotypes on particular genotypes of the host population [9]. Meanwhile, *B. graminis* has become a model organism to study host specialization of obligate biotrophic fungal pathogens.

With the dawn of next-generation sequencing, several studies provided initial insights in the haploid genomes of powdery mildews and the molecular basis of their obligate biotrophic lifestyle. Indeed, the first genomic studies, coupled with other “omics” approaches [10], showed that powdery mildews have experienced the loss of several, otherwise widely conserved Ascomycete genes with functions related to carbohydrate degradation, primary and secondary metabolism [11, 12], which could explain their strict dependence on live plant tissue. On the other hand, these genomes harbor an abundance of candidate secreted effector protein (CSEP)-coding genes, which were deemed to be crucial for successful pathogenesis [12, 13]. Isolate-specific variants of these powdery mildew CSEPs are recognized by matching intracellular immune receptors, encoded by barley or wheat *R* genes, which are present only in particular genotypes of these cereal hosts [9, 14, 15]. This demonstrates that at least these CSEPs are targets of the plant immune system and indicates co-evolutionary dynamics underlying interactions between the pathogen and cereal hosts at the population level. Genome sequencing of members of the cereal powdery mildew pathogen, *B. graminis*, led to the understanding that host specialization can occur by hybridization between two reproductively isolated *formae speciales* that multiply on different host species [16] and, possibly, also by “host tracking” or co-speciation [17, 18]. Comparative sequence analysis of multiple isolates of both barley and wheat powdery mildew pathogens, *B. graminis* f.sp. *hordei* (*Bgh*) and *B. graminis* f.sp. *tritici* (*Bgt*), revealed that at least their genomes are characterized by an ancient haplotype mosaic composed of isolate-specific DNA blocks, suggesting exceptionally rare outbreeding and dominant clonal reproduction of the haploid fungus in nature [12, 19].

Powdery mildew fungi have some of the largest genomes among plant-pathogenic Ascomycetes, strongly enriched with an unusually high content of transposable elements (TEs) [11, 12]. Extensive repetitive regions made up of TEs have hindered high quality short-read-based genome assemblies, resulting in severely fragmented datasets [6, 11, 12, 19]. The fragmentation of the available genomic assemblies has so far hampered our ability to address relevant biological questions, as for example the existence of long lineage-specific virulence regions, the impact of TEs on genome organization and evolution, as well as the conservation of gene order between diverged isolates.

In this study, we present a near-chromosome level assembly of the *Bgh* reference isolate (DH14), which recovers approximately 30 Mb of previously unassembled sequence, supplemented with a new, manually curated annotation. Genome-wide comparative analysis of the European DH14 isolate with the Japanese isolate RACE1, which is the most divergent *Bgh* isolate sequenced so far [9], revealed clear evidence for large-scale conservation of gene order between isolates. Subsequent comparisons with genomes of closely related *B. graminis formae speciales* indicated extensive copy number variation (CNV) of genes encoding secretion signal-containing proteins (SPs), which mirrors the phylogenetic relationships of these host-specialized forms. Finally, we found evidence for recent proliferation of TEs in the *Bgh* genome and possibly other *formae speciales* of *B. graminis*, but not in powdery mildews colonizing dicotyledonous host plants. Collectively, these genomic features reveal unprecedented insights into *B. graminis* life history and co-evolutionary patterns of the fungal pathogen with grass hosts.

## Results

### Large-scale *Bgh* genome organization

To facilitate a deep exploration of the *Bgh* genome, we applied third generation long-read DNA sequencing to generate high-quality genome assemblies of a European and a Japanese isolate, designated DH14 and RACE1. Whilst a short-read-based genome is available for DH14 [11], enabling direct comparison with the newly established long read-based assembly, isolate RACE1 was chosen because of its exceptionally high coding sequence divergence compared to a collection of 15 other *Bgh* isolates from different geographic origins, including DH14 [9]. Although the PacBio platform-based sequence depth for isolate DH14 was relatively low (~ 25×; Table 1), the Canu [20] assembly resulted in 963 contigs (i.e. 14,093 fewer contigs than the published reference genome), a significant increase of the N50 statistic (now 4.6 Mb), and an almost complete recovery of previously unassembled genomic sequences (Table 1). Using existing data from sequenced plasmid and fosmid clones [11], the assembly was further reduced to 318 scaffolds, comprising ~ 124.5 Mb in total. The final assembly was polished to remove erroneous base calls and insertions/deletions (indels) using short Illumina reads (~ 50× coverage). For isolate RACE1 the depth of the long read sequencing was higher (~ 50×) and thus these PacBio reads were used also for polishing. The resulting unscaffolded RACE1 assembly consists of 99 contigs (including the circular mitochondrial genome) and a total size of ~ 116.5 Mb (N50 3.9 Mb; Table 1). Overall, both assemblies show higher gene space coverage (BUSCO analysis) compared to the existing *Bgh* reference genome [11] although the difference is comparatively small (Additional file 1: Table S1). We did not find any evidence for the presence of previously reported *Bgh*-specific plasmid-like linear extrachromosomal DNA [21] in the two isolates.

**Table 1:** Assembly statistics for the genomes of the *Bgh* isolates DH14 and RACE1

	DH14 v3 (contigs)^a^	DH14 v3 (scaffolds)^a^	DH14 v4^b^	RACE1 v1
Assembly Statistics				
Number of sequences	15,056	6843	318	99
Minimum size	358	668	3069	16,042
1st Quartile	1206	1105	15,960	52,946
Median	1707	1254	23,353	358,063
Mean	5838	17,350	391,476	1,176,524
3rd Quartile	4940	1573	41,901	1,602,884
Maximum size	156,171	9,686,481	9,852,665	9,429,963
Total	87,906,467	118,726,170	124,489,486	116,475,897
N50	18,030	2,030,396	4,574,654	3,906,310
N90	1634	38,110	752,644	832,094
N95	1227	1521	57,430	443,704
Gap Statistics				
Number of gaps		4713	120	
Minimum size		26	110	
1st Quartile		425	1835	
Median		1710	4,44	
Mean		6524	5725	
3rd Quartile		6679	8,27	
Maximum size		36,100	24,554	
Total		30,749,686	687,104	
N50		27,231	9159	
N90		3672	2895	
N95		1997	2138	

^a^Genome version published by [11]

^b^Genome version generated in this study

To further assess assembly quality and to facilitate future genome-anchored genetic studies, we compared the assemblies with a previously generated genetic map for *Bgh* [22]. We located genomic positions for 80 mapped single-copy expressed sequence tag (EST) markers and compared their physical linkage with the corresponding genetic map. This revealed in most cases (67 out of 80 ESTs) a collinear marker order on physical and genetic maps (Additional file 2: Figure S1). In all but two cases in which discrepancies were found between physical and genetic maps, we observed collinearity of EST markers between the independently assembled genomes of DH14 and RACE1. Even large genetic linkage groups were mostly covered by only one or two genome contigs (e.g. linkage groups 2 to 7; Additional file 2: Figure S1), suggesting that our physical maps partly represent *Bgh* chromosomes or chromosome arms. In support of this, we identified 19 (DH14) and 20 (RACE1) cases of canonical telomeric repeat sequences (5′-TTAGGG-3′ hexamer; 34 to 61 tandem copies) at the beginning/end of contigs, in some instances together with distally-located gene-scarce regions likely resembling centromeres (Additional file 3: Figure S2, Additional file 1: Table S2). The gene-scarce regions are in all cases associated with specific long interspersed nuclear elements (LINEs) of the *Tad1* family (Additional file 3: Figure S2).

The circular mitochondrial genomes of both isolates were closed, yielding total sizes of 104 kb (DH14) and 139 kb (RACE1) (Additional file 4: Figure S3A), which is in agreement with older experimental estimates [23]. Nucleotide sequence alignment indicated > 96% identity of the mitochondrial DNA (mtDNA) of the two genomes. It further revealed that the RACE1 mitochondrial genome contains a ~ 32 kb duplication, while the DH14 mtDNA encompasses an ~ 1 kb isolate-specific sequence stretch that includes one predicted open reading frame (Additional file 4: Figure S3B). The structural and nucleotide differences might be linked to the isogamous and hermaphroditic manner of mitochondrial inheritance in *B. graminis* [24], meaning that the mtDNA can originate from two parents derived from distant *Bgh* populations. Nonetheless, the *Bgh* mitochondrial genome does not present major differences in gene repertoires compared to known mitochondrial genomes of other Leotiomycetes [25], except for *Atp9*, which encodes the subunit 9/c of the mitochondrial ATP synthase complex and has been transferred to the nuclear *Bgh* genome. Consistent with this, *Bgh Atp9* carries an N-terminal mitochondrial transfer signal sequence. This gene translocation has been observed in other fungal species and might be related to a physiological adaptation, enabling transcriptional modulation of its expression in cell- and tissue-specific contexts [26, 27].

### Identification of isolate-specific genes, gene duplications and gene expression

Existing *Bgh* gene models for the isolate DH14 were transferred to the new assembly and were supplemented by new predictions generated by MAKER [28], which were guided by protein and/or transcript evidence (whole-transcriptome shotgun sequencing; RNA-seq; see Materials and Methods). For RACE1, for which a prior genome annotation was unavailable, we generated de novo gene models using MAKER, guided by protein and transcript evidence from both *Bgh* isolates. We manually curated all gene models, removed poorly supported predictions, presumptive pseudogenes (mostly related to *Sgk2* kinase-like genes; [29]) and annotations that overlapped with TEs. During the manual curation we noted several instances of (tandem) duplicated genes, which are highly sequence-related at the nucleotide level, and thus had been collapsed into single gene models in the previous DH14 genome assembly [11]. This complicated the annotation and therefore new gene identification numbers (IDs) were generated also for DH14 (Additional file 1: Table S3).

The new annotation resulted in 7118 gene models for DH14, of which 805 genes encode predicted SPs. A similar number of 7239 gene models were found for RACE1 upon manual curation, including 770 that encode predicted SPs. A subgroup of SPs, called CSEPs, are secreted candidate virulence proteins defined by specific criteria [13]. For a more comprehensive coverage of the deduced fungal secretome, we generally included all SPs in our analyses. This also allowed us to incorporate newly detected effector candidates resulting from the re-annotation of the *Bgh* genome.

**Fig. 1:**
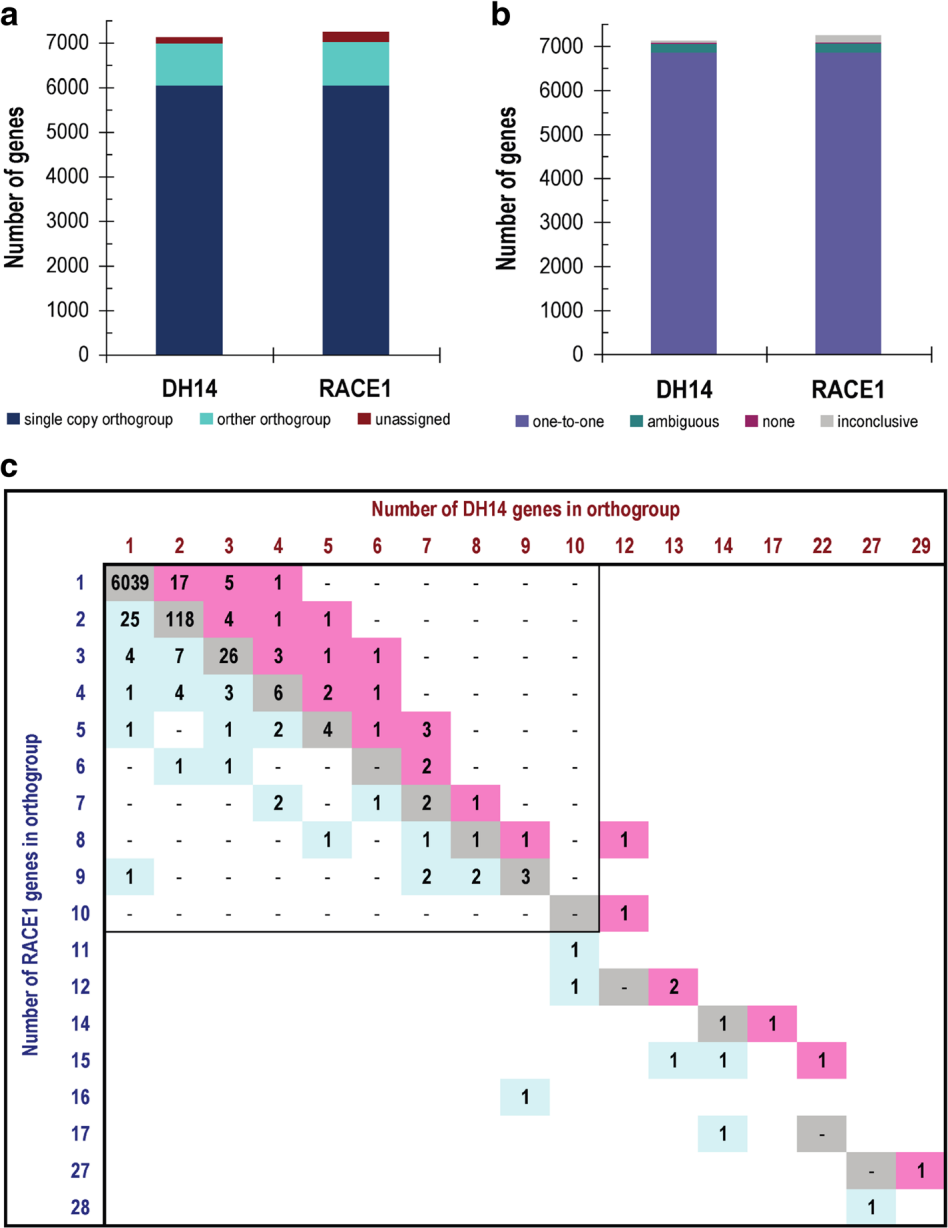
Identification of orthologous gene groups and gene pairs between the *Bgh* isolates DH14 and RACE1. **a** Bar graph visualizing the number of gene models in DH14 and RACE1 that were assigned by OrthoFinder into single copy orthogroups (with one member per isolate), orthogroups with more than two members, or with no ortholog at all. **b** Bar graph summarizing the observed orthology relationships between DH14 and RACE1 gene models, as inferred from a combination of OrthoFinder results and additional manual inspection of gene positions and synteny. With this method, a one-to-one relationship between isolates could be established for most genes (6844), while for ~ 200 genes in each isolate the relationship remained ambiguous (e.g. due to the existence of additional identical copies). Through further integration of RNA-seq data for both isolates, 31 and 27 genes were verified to be isolate-specific in DH14 and RACE1, respectively. For the remaining genes (47 in DH14 and 162 in RACE1) the relationship assignment was inconclusive due to still existing inaccuracies in the assemblies or annotations. **c** Graphical representation showing the composition of the identified orthologous gene groups from the OrthoFinder analysis. Most groups (6200 out of 6319) contain an equal number of members in DH14 and RACE1 (gray squares), while for some an isolate-specific enlargement can be observed with more members in one isolate than the other (light blue and pink squares)

To compare the gene repertoires encoded by the DH14 and RACE1 isolates, we first used OrthoFinder to infer orthologous gene groups (orthogroups). This analysis identified 6039 single-copy groups containing gene pairs with unambiguous one-to-one relationship between the isolates (Fig. 1a). By manually incorporating additional position and synteny information from a whole-genome alignment (see below) for the inference of orthologous gene pairs, we could further resolve some ambiguities and identify additional relationships for unassigned genes with more dissimilar sequences, significantly increasing the number of one-to-one gene pairs to 6844 (Fig. 1b, Additional file 1: Table S4). A comparison of DH14 and RACE1 orthogroups showed that most groups (6200 out of 6319) contain the same number of members in both isolates, but there are several groups with an isolate-specific expansion, indicating the presence of additional paralogs in one of the isolates (Fig. 1c, Additional file 1: Table S4). Such isolate-specific expansions occur almost 10-fold more frequently for SP-containing groups than for groups without SPs (9.4 and 1.2%, respectively; χ^2^-test, *p* < 2e^− 16^). An example for the occurrence of such an isolate-specific gene duplication is the *AVR*_*a1*_ avirulence effector [9] for which two identical copies exist in DH14, while only one copy was found in RACE1 (Additional file 5: Figure S4A). By contrast, the *AVR*_*a13*_ avirulence effector locus is highly similar in both isolates, with a single copy of *AVR*_*a13*_ flanked by the other two members of the previously identified *AVR*_*a13*_ *CSEP* family [9, 13] (Additional file 5: Figure S4B).

We also searched for isolate-specific genes without any related sequence in the other respective isolate. For this purpose, we included the previously published RNA-seq data for RACE1 [9] and a corresponding newly generated dataset for DH14 as additional evidence to extract a high-confidence set of isolate-specific genes. This analysis identified in total 31 isolate-specific genes in DH14, for 13 of which we detected credible gene expression (FPKM (fragments per kilobase [sequence length] and million [sequenced fragments]) ≥ 5) during pathogenesis (Additional file 1: Table S5). A similar number of 27 isolate-specific genes was found in RACE1, of which 19 were also expressed (FPKM≥5) during pathogenesis (Additional file 1: Table S5). Among these expressed isolate-specific genes, we found eight *SPs* in DH14, but only three in RACE1. As the two isolates are of opposite mating types, also the corresponding *MAT* idiomorphs were among the isolate-specific genes, with DH14 carrying *MAT1–2-1* and RACE1 carrying both *MAT-1-1-1* and *MAT-1-1-3* (Additional file 1: Table S5, Additional file 6: Figure S5).

Apart from a validation of the presence of isolate-specific genes, the RNA-seq data enabled us to examine also potential isolate-specific gene expression during infection. We searched for orthologous gene pairs for which we could detect robust transcript levels (FPKM≥10) in one of the two isolates while in the other the corresponding gene was not expressed (FPKM< 1 or raw count≤2), and for which isolate-specific expression could be further validated by visual inspection in the Integrative Genomics Viewer (IGV; [30]). A total of 15 genes showed differential expression based on these criteria (Additional file 1: Table S6). Of these genes, 12 were specifically expressed in RACE1 (of which seven encode CSEPs), while three were expressed specifically in DH14 (Additional file 1: Table S6), indicating manifest differences in expressed gene repertoires between *Bgh* strains.

### Genome synteny, structural and sequence variation between isolates

**Fig. 2:**
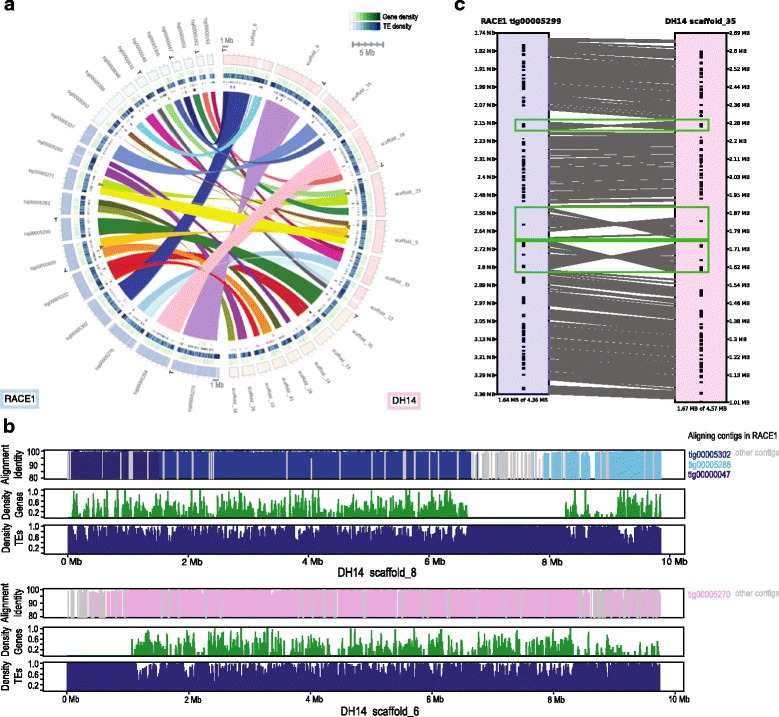
Syntenic relationships and genomic rearrangements between the genomes of *Bgh* isolates DH14 and RACE1. **a** Circos diagram illustrating the syntenic relationships between the genomes of DH14 and RACE1. Based on the corresponding N50 values, the 8 and 11 largest scaffolds/contigs from the DH14 and RACE1 genomes and the corresponding aligning scaffolds/contigs were chosen for visualization. Syntenic regions were identified based on whole-genome alignment, and aligning regions (≥2 kb, similarity ≥75%) are connected with lines. The surrounding circles represent from the outside: on the right DH14 scaffolds (pink) and on the left RACE1 contigs (blue), with all unaligned regions (≥ 1 kb) indicated as white gaps; gene density (green) and TE density (blue) calculated in 10 kb sliding windows; locations of all genes predicted to code for SPs; locations of isolate-specific genes coding for SPs (dark red) or any other proteins (black) and isolate-specific additional gene copies/paralogs coding for SPs (pink) or any other proteins (grey). The arrowheads indicate observed alignment breaks of at least two large-scale genomic rearrangements (green and brown arrowheads, respectively). Scaffolds or contigs marked with “T” indicate presence of telomeric repeats. **b** Visualization of sequence identity to RACE1, gene and TE density along the two largest DH14 scaffolds. The top panel shows the sequence identity to the aligning RACE1 contigs obtained from the whole-genome alignment, with bars colored according to the involved RACE1 contig as indicated next to the graph. The two lower panels show the gene density (green) and TE density (blue) in DH14 calculated in 10 kb sliding windows along the DH14 scaffolds. **c** Detailed synteny between RACE1 tig00005299 and DH14 scaffold_35 visualized with SyMap [87]. Local inversions within the otherwise syntenic region are highlighted by green boxes, while grey lines connecting the RACE1 and DH14 contigs represent the syntenic alignments and black marks on the contigs indicate the positions of annotated genes

For a detailed genomic comparison, we conducted a whole-genome alignment of DH14 and RACE1 assemblies using MUMmer [31]. Although RACE1 was chosen for genome sequencing based on its high sequence divergence to DH14 within coding regions [9], the genomes of the two isolates overall are still remarkably similar, with 92 and 98% of the assemblies of DH14 and RACE1 aligning to the corresponding other isolate at an average nucleotide sequence identity of ~ 99%. Moreover, the aligned sequence stretches form large syntenic blocks of up to 9 Mb, implying that gene order within the assembled contigs is also largely conserved between the isolates (Fig. 2a). A closer inspection of the syntenic blocks revealed that the large-scale synteny between DH14 and RACE1 can be interrupted locally by intermittent stretches of non-syntenic alignments (e.g. to a different contig in the other isolate) or by sequence areas without a close match in the other genome (Fig. 2b). These interspersed alignment gaps typically are rather small (< 1 kb on average) and concern primarily regions of repetitive sequence, while only rarely affecting protein-coding genes (only 1% of alignment gaps affect genes). As both genomes are not resolved entirely to whole-chromosome level, we cannot estimate the full extent of large-scale chromosomal reshuffling. Nevertheless, the occurrence of within-contig alignment breaks provides evidence for at least two large-scale genomic rearrangements that involve genome stretches larger than 1 Mb (Fig. 2a, Additional file 7: Figure S6). Additionally, we found 128 cases of genomic rearrangements within contigs, where sequence stretches of at least 10 kb are inverted relative to the other isolate. These inversions occur dispersed throughout the genome and the average size of inverted regions is around 20 kb with only seven regions larger than 50 kb. Roughly half of these local inversions (69 out of 128) affect gene-containing regions, but only for 22 of the corresponding regions we could verify by manual screening that they coincide with an inverted gene order relative to the flanking genes (Fig. 2c, Additional file 1: Table S4). In three of these cases, a further re-shuffling of genes was observed within the inverted region. Collectively, while large parts of the genome structure and gene order seem to be well conserved, we detect a number of mostly smaller synteny breaks that are dispersed throughout the genome and contribute to the structural variation between the two isolates.

To examine the sequence variation between RACE1 and DH14, we used the single nucleotide polymorphisms (SNPs) identified in the MUMmer [31] dnadiff analysis to calculate SNP frequency in 10 kb sliding windows. In addition, we obtained SNPs for isolates A6 and K1 [19] from a short-read-based alignment (see Methods) and calculated the corresponding SNP frequencies to re-examine the sequence variation of these isolates to the improved DH14 reference genome. On average, the overall SNP frequency is three times higher in RACE1 (4.7 SNPs/kb) than in A6 (1.4 SNPs/kb) and K1 (1.3 SNPs/kb). Moreover, a comparison of SNP frequency distributions between the three isolates shows that in RACE1 SNP frequencies below one SNP per kb are seen only rarely, whereas in A6 and K1 they are common (Additional file 8: Figure S7A). Accordingly, a two-component mixture model fitted to the observed SNP frequencies recovered the previously described [19] distinction between low and high SNP densities (mean ± standard deviation) for A6 (low: 0.1 ± 0.1; high: 1.9 ± 1.5) and K1 (low: 0.1 ± 0.1; high: 2.0 ± 1.7). By contrast, for RACE1 no such distinction could be observed and the SNP frequency was high for both model components (low: 3.8 ± 2.4; high: 10.6 ± 6.1; Additional file 8: Figure S7B).

### *SP* paralogs typically reside in close proximity

Although local clustering – in part even as tandem duplicates - of genes encoding effector candidates in the *Bgh* genome has been suggested and described [13], its scale at a genome-wide level remained unclear. This is mainly due to the severe fragmentation of the previously available genomic assemblies and the collapse of highly similar gene copies in the short-read-based assemblies [11, 19]. We therefore explored systematically whether *SPs* in general co-occur in close distance. Here we defined *SP* clusters based on two criteria: (1) each cluster contains at least three *SPs* and, and (2), two *SPs* are separated by a maximum of ten genes coding for non-secreted proteins. By these criteria, 72% of the SP-coding genes (583 out of 805) can be placed in three large clusters with more than 30 *SPs* and 74 smaller clusters with less than 20 *SPs* (Additional file 1: Table S7). Consistent with an earlier study [13], many of these clusters harbor sequence-related genes which belong to the same orthogroup (Additional file 1: Table S7), suggesting that they might originate from recent local duplications with subsequent sequence diversification, thus likely representing paralogs. Despite this occurrence of *SP* clusters, we did not observe local enrichment of *SPs* on particular genomic scaffolds (Additional file 9: Figure S8A). Rather we found that the *SP* count follows the scaffold size (Additional file 9: Figure S8B), which is in line with the results of a χ^2^-test that did not detect a significant deviation between the *SP* frequency per scaffold and the underlying genome fraction per scaffold (*p* = 0.21).

### Copy number variation of *SPs* within and between *formae speciales* correlates with phylogeny and host specialization

To investigate the extent of within-genome gene duplications we used MCScanX [32] on the DH14 and RACE1 isolate datasets. As expected for a haploid genome, the majority of the genes exist in single copies, but ~ 10% have one or more paralogs (Additional file 1: Table S8). Approximately one third of these duplications occur in tandem (36%), while the remaining ones are either proximal (in-between the next five genes) or dispersed throughout the genome (30 and 33%, respectively). When compared to the genomes of the phylogenetically closely related phytopathogenic fungi *Botrytis cinerea* and *Sclerotinia sclerotiorum*, the *Bgh* genome shows a higher percentage of duplications (11% versus 0.3 and 5.4%, respectively). A closer look at the *S. sclerotiorum* dataset revealed that the seemingly elevated number of dispersed and proximal duplications in this species is mainly comprised of retrotransposases that are retained in the corresponding annotation (Additional file 1: Table S9). This finding indicates that the comparatively high number of paralogous gene pairs (812 out of 7118) in the *Bgh* genome is a unique characteristic among the so-far-sequenced Leotiomycetes.

We investigated whether these duplications can be associated with certain types of genes or functional domains and found that *SP* genes are significantly more subject to duplication than genes encoding non-SPs (χ^2^ test, *p* < 0.001). Most duplications of *SPs* seem to occur in tandem (Additional file 1: Table S10). Functional domain associations are poor for the group of *SP* genes because effector proteins often have few or no functional descriptions (applies to ~ 79% of the 805 predicted SPs in DH14 in PFAM-based searches; Additional file 1: Table S11). However, there are two clusters with tandemly duplicated genes that are rich in genes encoding ribonuclease-like domains (SUPERFAMILY SSF53933, clusters 21 and 1), and two clusters with *Egh16* virulence factor homologs (PFAM PF11327, clusters 56 and 14). Among the genes coding for non-SPs, a portion of the duplications (181 out of 546) are related to genes with kinase-like domains (SSF56112, PS50011), which have been described previously as an over-proliferating family in the *Bgh* genome [29].

**Fig. 3:**
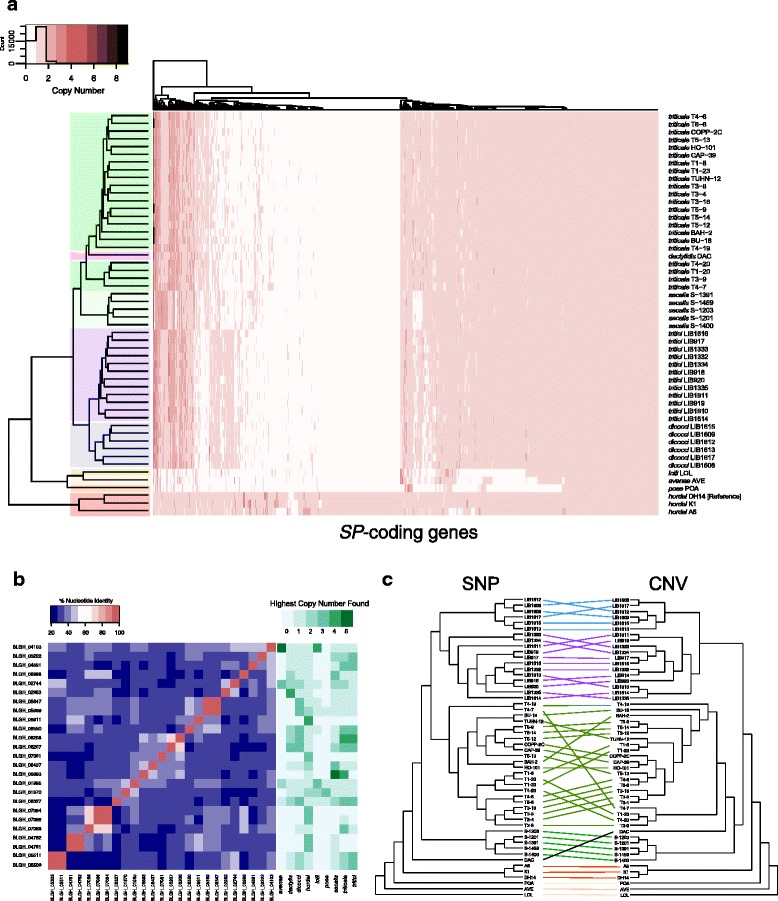
CNV of *SPs* reflects the phylogeny of various *formae speciales* and their isolates. **a** Heatmap illustrating the *SP* copy number per individual genome of various *B. graminis formae speciales* (*avenae*, *dactylis*, *dicocci*, *hordei, lolii*, *poae, secalis*, *triticale* and *tritici*) based on read mapping depth. Hierarchical clustering (Euclidean method) of the *SPs* and the isolates based on the CNV values is shown on the left and above the figure, respectively. Genomes belonging to the same *forma specialis* are color-highlighted on the left dendrogram. **b** Nucleotide identity matrix of high copy number *SP* subset (> 3 in at least one isolate, excluding BLGH_02064, BLGH_02719, BLGH_07048 due to ambiguities in the gene model) resulting by alignments with EMBOSS-Needle [106]. On the right, the highest copy number found in each *forma specialis* for these *SPs* is depicted. **c** Comparison between the hierarchical clustering dendrogram derived from the CNV analysis (Euclidean method) and the SNP-based UPGMA cladogram generated with SplitsTree [105] using a tanglegram generated with Dendroscope [107]. Lines connect the same isolate, while colors correspond to different *formae speciales* (as in A)

Based on the above results we sought to determine whether gene copy numbers vary between strains belonging to different *formae speciales* of *B. graminis*. Using published datasets [12, 16, 18, 19], we estimated the copy number of each *SP* based on the observed coverage of short-read-based sequence alignments against the DH14 assembly (Fig. 3a). To assess the accuracy of this analysis, a sample of genes with tubulin or actin functional domains and some additional non-SP-coding genes with conserved domains was used. As expected, this control dataset showed minimal variation and revealed conserved single-copy presence in all 52 genomes examined (representing 9 *formae speciales*; Additional file 10: Figure S9), indicating that the analysis based on coverage depth is robust. Nonetheless, false approximations cannot be fully excluded by this approach.

For the majority of *SPs* (458 of 805; 57%) we detected simple presence/absence variation between the different *formae speciales* and their respective isolates (Fig. 3a). For a smaller fraction of *SPs* (201 of 805; 25%) the number of observed copies varies between 0 (absence) and more than 2 copies per genome. Interestingly, while variation in copy numbers between the examined genomes can be observed (Fig. 3a), for many *SPs* (72–87%, depending on the *forma specialis*) the number of gene copies is conserved among different isolates of the same *forma specialis* (e.g. BLGH_01048 has 2 copies in all f.sp. *secalis* and f.sp. *dicocci* genomes). In addition, high-copy *SPs* have low sequence similarity with each other (Fig. 3a). To investigate whether CNV correlates with the phylogeny of the *formae speciales*, we generated a tanglegram using a dendrogram derived from the hierarchical clustering of the CNV data and a cladogram derived from a UPGMA tree based on ~ 1.07 million single nucleotide polymorphism (SNP) positions between the isolates (Fig. 3a). The CNV-based dendrogram accurately groups the isolates based on their host specificity, indicating that isolates belonging to the same *formae speciales* have distinctive CNV profiles.

### The *Blumeria* core effectorome

To define the core effectorome of the species *B. graminis*, we de novo-assembled and annotated the genomes of single isolates of the 9 *formae speciales* and inferred orthology relationships for the predicted proteomes*.* Our amino acid sequence-based orthology clustering of the predicted *SPs* (Additional file 11: Figure S10A) suggests that although part (see below) of the secretome is highly conserved in all *formae speciales*, another subgroup is divergent. Also, due to the divergence at the DNA sequence level the presence of certain *SPs* in the genomes of the more distantly related *formae speciales avenae*, *lolii* and *poae* was not detectable in the short-read-based CNV analysis above (Fig. 3a), while the orthology analysis identified related sequences at the amino acid level (Additional file 1: Table S11). Yet, the *formae speciales avenae*, *lolii* and *poae* still share smaller intersections with the *Bgh* secretome compared to the rest (Additional file 10: Figure S10A).

Out of the 805 *Bgh SPs* present in isolate DH14, 442 have at least one ortholog in all genomes assayed. A considerable fraction of these widely conserved *SPs* (252 out of 442; 57%) has PFAM domains and/or homologs outside the *Blumeria* genus. As indicated by their functional annotation (e.g. peptidases/proteases, hydrolases), these SPs are rather part of a common SP repertoire of fungal plant pathogens and are not specific innovations of the grass powdery mildews. On the other hand, 190 SPs fulfil the typical CSEP criteria (no homology outside the Erysiphales*,* no PFAM domain; [13]) and can be considered as the core effectorome of the grass powdery mildews. These core CSEPs belong to different phylogenetic families (Additional file 10: Figure S10B), possibly targeting a core set of conserved host functions to maintain virulence on grasses.

### The *Bgh* genome exhibits no obvious compartmentalization

**Fig. 4:**
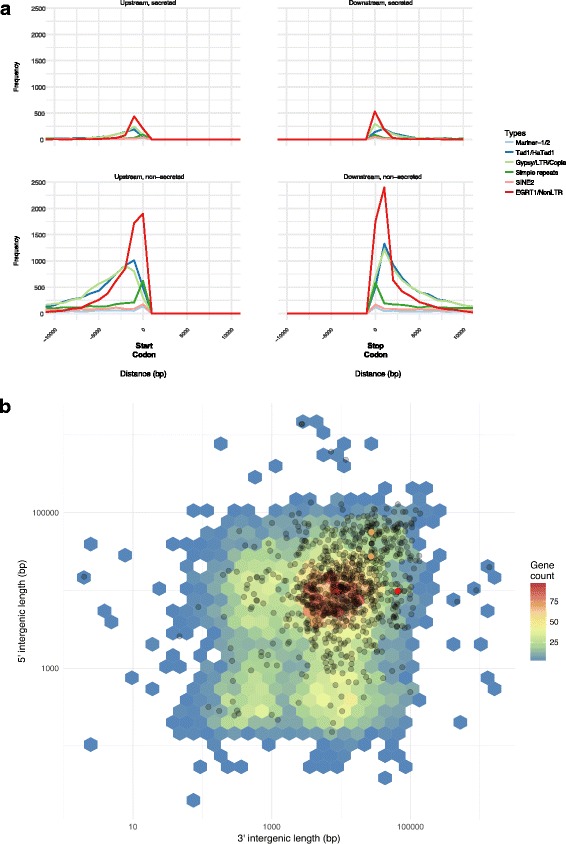
Intergenic space has similar size and is occupied by TEs in case of both *SPs* and non-*SPs.* **a** Frequency plot of the distance (− 10,000 to + 10,000 bp) of repetitive elements from the start codon (left panel) or the stop codon (right panel) of *SPs* (top panels) and non-*SPs* (bottom panels). The lines are color-coded and each represents a class of TEs according to the given legend. **b** 5′ and 3′ intergenic space size (y and x-axis) was calculated using BEDTools on the DH14 reference annotation. Black dots depict the intergenic length of all *SPs*, colored hexagons indicate the intergenic length of all non-*SPs.* Note the color-code indicating the frequency distribution (gene count according to the legend on the right) of non-*SPs*. The orange dots mark the two *AVR*_*a1*_ copies and the red dot marks *AVR*_*a13*_

Various types of TEs that are dispersed more or less evenly throughout the genomes (Fig. 4a, Additional file 1: Table S11) dominate the intergenic space of the DH14 and RACE1 genomes. Accordingly, many TEs can be found in close vicinity to genes, regardless if they are coding for SPs or not (Fig. 4a). This pattern contrasts with other sequenced fungal and oomycete plant pathogens where transposon-rich areas are essentially limited to lineage-specific regions/chromosomes or are largely confined to isochores [33, 34]. Several copies of these elements seem to be expressed, in particular certain types of *Copia* elements (Additional file 1: Table S12), and in many cases, overlap with the 5′ or 3’ UTRs of nearby genes (Fig. 4a).

A complementary analysis of the local gene density, measured as flanking distances between neighboring genes, shows that in general the flanking distances in the *Bgh* genome are rather high, with an average distance of ~ 14 kb (Fig. 4b). Accordingly, the surrounding genomic context of most genes in the *Bgh* genome is gene-sparse and repeat-rich and large flanking distances are not specific to *SP* genes (Fig. 4b). In line with this pattern, also the flanking distances of the two known *Bgh AVR* effector genes, *AVR*_*a1*_ and *AVR*_*a13*_ [9], are not exceptionally large compared to the overall genome (Fig. 4b). We further investigated whether genes coding for CSEPs or other SPs, which do not fulfil the typical effector criteria, present a difference in their 5′ or 3′ intergenic distances compared to ascomycete core ortholog genes (*COGs*). Regarding the 5′ intergenic distances, we detected no marked variation between the groups (ANOVA, *p* = 0.382), while the 3′ intergenic distances on average were slightly larger for the *COGs* than for both the *CSEPs* and other *SPs* and (ANOVA, *p* = 0.004; Tukey post hoc tests, *p* < 0.05; Additional file 3: Figure S2B). The results of this analysis highlight that in *Bgh CSEPs* or other *SPs* are not located in peculiar gene-scarce regions compared to the conserved *COGs*. In addition, low gene density also could not be associated with high dN/dS rates (Additional file 3: Figure S2C), indicating that fast evolving genes in *Bgh* such as the *CSEPs* can occupy both gene-rich and gene-scarce areas. Thus, the *Bgh* genome does not appear to be split into distinct compartments, but is rather characterized by a low gene density and high TE density throughout the entire genome.

### A recent lineage-specific TE burst shaped the *Bgh* genome

Since TEs occupy the majority of the *Bgh* genome and are in many cases closely entangled with presumed virulence genes (*SPs*), we examined whether these repetitive sequences slowly accumulated over time or, alternatively, were subject to sudden expansions in the life history of powdery mildews, which might be linked to the observed proliferation and clustering of some highly sequence-related *SPs*. We used RepeatMasker (www.repeatmasker.org) to detect TEs with previously curated annotations found in Repbase (http://www.girinst.org/about/repbase.html), and subsequently generated repeat landscapes based on the divergence from the corresponding consensus TE sequences.

**Fig. 5:**
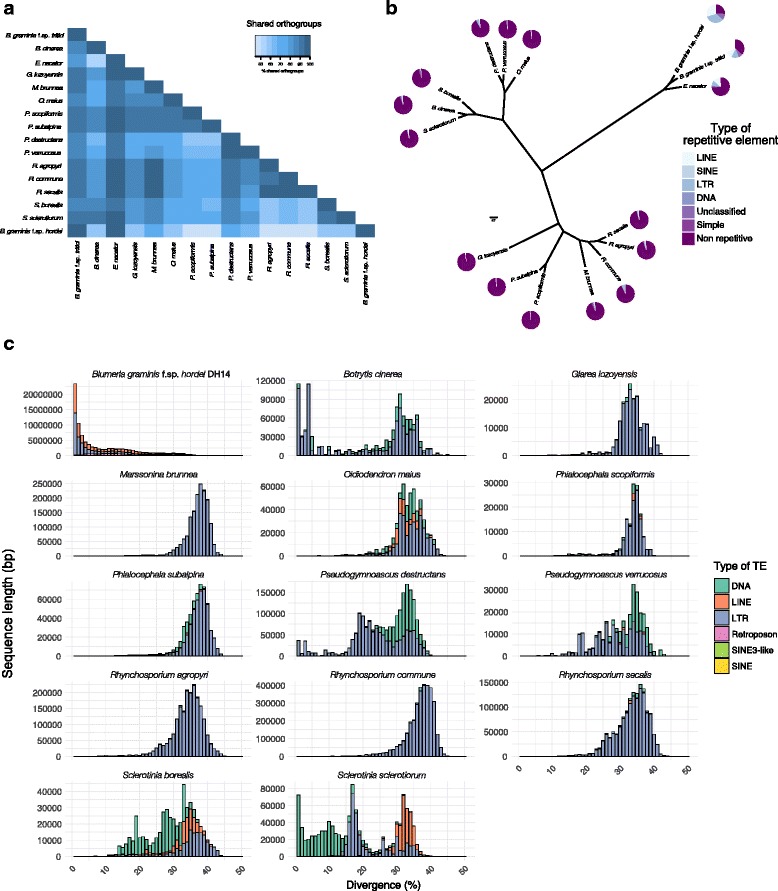
A recent TE burst in *Bgh*. **a** Orthogroup relationships of Leotiomycete genomes. Leotiomycete genomes with available annotations in the NCBI database were assayed for their relatedness to the *Bgh* genome using OrthoFinder [82]. Percentage of shared orthogroups is color-coded according to the key above (abbreviations of the species used can be found in Additional file 1: Table S13). **b** Multi-locus phylogeny for 16 Leotiomycetes generated from 2639 single copy orthologous genes identified with OrthoFinder [82] and drawn with FigTree (http://tree.bio.ed.ac.uk/software/figtree/). The repeat content, type and the portion it represents in each species’s genome is depicted in pie charts at the tip of each branch. The scale bar indicates the number of amino-acid substitutions per site. **c** Repeat landscapes for each genome of the tree shown in (**b**). Plots for *B. graminis* f.sp. *tritici* (BGT) and *E. necator* (ERYN) can be found in Additional file 10: Figure S9. The sequence length occupied in the genome is depicted on the y-axis (not normalized among the species examined), while the percent divergence from the corresponding consensus sequence is given on the x-axis

Surprisingly, most of the repetitive elements in *Bgh* show very low nucleotide sequence divergence (< 10%) compared to the TEs in 13 closely related Leotiomycete genomes (typically 30–40% nucleotide sequence divergence; Fig. 5a, b), suggesting a recent lineage-specific expansion of TEs within *Bgh* (Fig. 5b, c). In addition, there are 1866 occurrences of long terminal repeats (LTRs) with less than 0.1% divergence associated with either *Gypsy* or *Copia* elements (~ 3% of the LTRs than can be identified), indicating that the *Bgh* genome carries very recent transposition events. Finally, the observed TE expansion in *Bgh* can be equally attributed to both LINE and LTR retrotransposons (Fig. 5c). As outlined above, for part of these TEs, in particular *Copia* elements, evidence of expression can be found in the RNA-seq datasets (Additional file 1: Table S12).

Genome assemblies of *B. graminis* isolates belonging to other *formae speciales*, which are exclusively based on short reads, were found to underestimate both the magnitude of TE expansion and the presumed divergence time. This is due to the fact that the majority of the highly similar repetitive sequences collapse into few contigs, as revealed by the comparison of *Bgh* assemblies that are either based on long (PacBio) or short reads (Illumina; Additional file 12: Figure S11A). Therefore, for the other *formae speciales* of *B. graminis* it can only be assumed that they also experienced a recent TE expansion, while it remains unclear whether this event is older or more recent than the one in *Bgh*.

Remarkably, when applying the same pipeline on the sequenced genomes of the dicot-infecting powdery mildews *Erysiphe necator*, *E. pisi* and *Golovinomyces orontii*, the divergence from the consensus of the respective TE sequences is much higher (25–35% compared to < 10% in *Bgh*), suggesting that the expansion of the repetitive elements in these species is more ancient than in *Bgh* (Additional file 12: Figure S11B). This calculation is unlikely to be an underestimation due to the short-read-based genome assemblies of these species as long-(PacBio) or short-read-based assemblies revealed similar divergence rates for *G. orontii*. Because evolutionary rates within the *Erysiphaceae* family appear to be comparable [35, 36] and essentially all TEs in dicot-infecting powdery mildews are sequence-diverged (> 10%; Additional file 12: Figure S11B), this observation suggests independent “transposon bursts” for each powdery mildew lineage that occurred at different times.

## Discussion

### An improved assembly provides insights into large-scale organization of the *Bgh* genome

The genome of the obligate biotrophic pathogen *Bgh* is characterized by a loss of genes encoding enzymes of primary and secondary metabolism as well as an expansion of overall genome size due to a massive proliferation of TEs [11]. This high repeat content, with TEs representing more than two thirds of the genome, makes it essentially impossible to generate chromosome-level DNA assemblies from short sequencing reads that do not allow to resolve these highly similar sequences. Accordingly, the first short-read-based assembly for *Bgh* isolate DH14 was highly fragmented, with more than 15,000 contigs (and close to 7000 scaffolds), and about one third of the estimated genome size was not covered [11], possibly due to collapsed sequences.

We here used a single-molecule sequencing technique to generate long DNA sequence reads, which enabled us to establish high quality genome assemblies for the two *Bgh* isolates, DH14 and RACE1. For DH14, this long-read-based assembly showed a > 10-fold improved contiguity and recovered a substantial amount of previously unassembled genomic sequence (50% increase in genome size) compared to the first genome draft. In both DH14 and RACE1 assemblies, the largest contigs are more than 9 Mb in size, likely to represent complete chromosome arms, and a similar number of observed telomeric repeat regions in both assemblies (19 and 20) suggests the *Bgh* genome is likely partitioned into 10 chromosomes.

The high assembly quality is also supported by both an improved gene space coverage (now > 98% BUSCO coverage for the newly annotated DH14 reference genome; Additional file 1: Table S2) and good agreement with a previously published genetic map of *Bgh* [22]. The yet missing BUSCOs could be due to either real gene loss events or failure to detect the corresponding conserved ortholog by the software, suggesting that the core gene space is now essentially completely covered in the *Bgh* reference genome. The few observed discrepancies between physical contigs and the genetic map might be attributed to the fact that the linkage map was constructed from a cross between two isolates (C15 and JEH31) that are different from the ones used in this work. Therefore, while we cannot exclude that the few discrepancies are at least partly due to remaining inaccuracies in either our assemblies or the genetic map, these also could be evidence for additional isolate-specific genomic rearrangements.

### Effector repertoires differ slightly between the two *Bgh* isolates

Approximately 74% of both assemblies are made up of repetitive elements that are uniformly dispersed across the genome, which is an even higher repeat fraction than predicted before for *Bgh* (64%; [11]). The previous underestimation of the TE content could be due to the collapse of highly repetitive short-read-based sequences during genome assembly. While a comparable fraction of TEs was described for the oomycete pathogen *P. infestans* [37], other sequenced fungal genomes contain markedly lower fractions (Fig. 5b; [38]).

In addition to a drastically improved assembly of repetitive sequences, we also noticed the existence of loci with similar or identical copies of a number of genes, which had previously been collapsed into single gene models (Additional file 1: Table S3). Thus, genome re-annotation based on the new assemblies also provided an improved representation of *Bgh* gene repertoires, increasing the number of gene models from 6470 [11] to 7118 in the annotation of the reference isolate DH14. A subsequent comparison of protein-coding genes between DH14 and RACE1 revealed largely conserved gene numbers between the two isolates, which is in agreement with previous observations based on short-read-based assemblies of three *Bgh* isolates [19]. However, due to the improved resolution, here we were able to identify several cases of isolate-specific gene family expansions and gene duplications, especially affecting *SPs*. Moreover, we identified several *SP* genes that were present exclusively in one of the genomes and lacked any similar sequence in the other isolate. The observed differences suggest that diversity of *SP* repertoires in *Bgh* is maintained mostly through gene duplications with subsequent sequence diversification and gene deletions. Thus, our observations for *Bgh* reflect the general evolutionary pressure on pathogen populations to diversify effector repertoires, which could then serve as reservoirs for rapid adaptation in response to population-level alterations in host *R* genes. Accordingly, the diversity of *Bgh* effectors is important in balancing the trade-off between ensuring virulence function and, at the same time, trying to escape detection by the host plant [39].

Interestingly, the gene with the strongest isolate-specific expression in RACE1 encodes thioredoxin A, which is important for protection from oxidative stress and contributes to virulence of human pathogenic bacteria and fungi [40, 41]. However, as the RNA-seq samples for RACE1 and DH14 were generated in different experimental batches, we cannot fully rule out the possibility that the expression differences we observed could be partially influenced by batch effects.

### TEs and *SPs* are evenly dispersed throughout the genome

Many filamentous pathogens exhibit a distinct genome architecture, denoted as “two-speed genome”, with well-defined blocks of low gene and high TE density, interspersed between the generally more prevalent genomic areas of high gene and low repeat content [42–44]. These TE-rich blocks, which often harbor genes encoding secreted effector proteins, typically exhibit high lineage-specific diversity and are prone to be involved in genomic rearrangements [42, 43, 45]. In this way, these regions are thought to provide a pool of genetic variation that is needed by phytopathogens to quickly adapt to changing requirements in the evolutionary arms race with their hosts [44].

In *Bgh*, however, the situation is clearly different, as the numerous TEs are not restricted to specific areas, but rather evenly dispersed throughout the genome (Fig. 2a, Additional file 9: Figure S8A). In addition, neither are the flanking regions of *SPs* particularly enriched in TEs, nor are they markedly larger compared to non-*SPs* (Fig. 4a, b). Moreover, *SPs*, whether they are categorized as putative effectors (CSEPs) or not, are not associated with unusually gene-sparse (Additional file 3: Figure S2B) or peculiar genomic regions (Additional file 9: Figure S8A), but their number is positively correlated with scaffold size (Additional file 9: Figure S8B). Additionally, the dN/dS ratio of the *CSEPs* is not associated to local gene density (Additional file 3: Figure S2C).

We also did not detect any large lineage-specific regions as reported for *Verticillium dahliae* [43]. Instead, smaller lineage-specific (< 1 kb on average; up to 51 kb) or locally inverted (< 20 kb on average; up to 90 kb) sequence stretches can be found dispersed rather evenly distributed throughout the genomes of the two *Bgh* isolates. Thus, the organization of the *Bgh* genome does not match the “two-speed genome” model [44], in which genetic variation is concentrated in specific genomic areas. Instead, *Bgh* appears to have a “one-speed/high-speed genome” where genetic and structural variation is not tied in specific compartments but rather sustained throughout the whole genome. Such a genome architecture might contribute to maintaining genetic diversity of mainly asexually reproducing *Bgh*. However, in this scenario genomes would be expected to rapidly lose the overall synteny due to TE activity and cumulative effects of local genome rearrangements. Thus, it is conceivable that occasional sexual reproduction ensures the maintenance of overall synteny of *Bgh* genomes.

Our present work supports the assumption of a predominantly asexual reproduction mode in *Bgh*, as we were able to recover the previously described mosaic genome structure in the European *Bgh* isolates (A6, K1 and DH14), with isolate-specific alternating regions of low and high sequence diversity [19] (Additional file 8: Figure S7). For the highly divergent Japanese isolate RACE1, on the other hand, no monomorphic regions were detectable relative to DH14 (Additional file 8: Figure S7), which is most likely due to the prolonged geographic separation of the two isolates during which sequence variation could accumulate at a whole-genome scale.

### Grass powdery mildews have a fast-paced secretome adapted to their respective hosts

Effector proteins play a crucial role in interactions between plant pathogens and their respective hosts [46], and consequently both small (sequence divergence) and big (loss of effector clusters) changes can drive the preference of the pathogen to a new host [47]. To date, several genome reports have established that phylogenetically related pathogens share a core effectorome, whereas each member of a taxonomic lineage contributes its own unique effectors to the pan-effectorome [48–50]. The large number of candidate effectors in the core effectorome of the *Blumeria* genus identified here, including at least 190 CSEPs belonging to 74 gene families, suggests these are indispensable for the maintenance of fungal virulence on different monocotyledonous hosts in each *forma specialis* of the species *B. graminis*. Whether the corresponding effector families mainly target different host components belonging to few or a large number of cellular pathways for the establishment of a biotrophic relationship with their grass hosts remains to be tested.

One interesting aspect of the grass powdery mildew effectorome is the CNV that some of its members experience (Fig. 3a, b). As in other plant pathogens, this variation is dominated by presence/absence polymorphisms [49], indicating strong selection for some *SPs* by certain host genotypes. In addition, we noted increased numbers of *SP* copies in particular isolates and *formae speciales*, suggesting that transcript dosage might also play a role in host adaptation of powdery mildews. In other plant pathogens, increased copy number of virulence genes can alter the infection phenotype, as for example reported in the case of *ToxB* in *Pyrenophora tritici-repentis* [51, 52].

For powdery mildews this evolutionary pattern might be particularly advantageous because the loss of the repeat-induced point mutation mechanism (RIP; [11]) allows additional gene copies to remain intact and functional [53], providing a presumed fitness advantage. This can be for example observed in *Erysiphe necator*, where an increased copy number of *EnCYP51* enhances fungicide resistance [6]. Similarly, careful re-examination of existing data using the information from the new *Bgh* reference assembly indicates that for some isolates duplications of *CSEPs* could offer the means to escape detection by their respective host via naturally accumulating mutations in one of the copies. An example of this might be *AVR*_*a1*_, where one of the two copies present in isolate CC107 has accumulated mutations allowing evasion of detection in barley cultivars carrying the matching *Mla1 R* gene [9]. A recent report [54] suggests extensive *forma specialis*-specific expansions of certain *CSEP* families, supporting the conclusions of our CNV and *SP* orthology analysis (Fig. 3a, Additional file 11: Figure S10A).

### TEs expanded suddenly and massively in the *Erysiphaceae*

The *Bgh* genome is frequently referred to as a typical example of repeat-based expansion of an eukaryotic genome [55]. Even early studies predating high-throughput genome sequencing revealed that the effect of TEs in this pathogen’s genome is significant. This conclusion was based on the frequency these sequences are associated with coding regions [56–58]. Nevertheless, the question of whether the activity of TEs and their dominance in the genome has been a beneficial or a neutral feature is still open.

TEs in the genome of *Bgh* are evenly distributed, in part transcriptionally active and flank virulence genes as much as genes involved in all types of basic biological processes (Fig. 2a, Additional file 3: Figure S2, Additional file 9: Figure S8A). As in many other cases, it can be hypothesized that TEs can act as templates for rearrangements, deletions and duplications of genomic sequences [42, 49]. Furthermore, TE insertions next to or within virulence genes can change the pathogen’s host range [59, 60].

We show for the first time that TEs in the grass-infecting (*Blumeria*) and dicot-infecting (*Erysiphe*) powdery mildews experienced sudden and, in evolutionary terms, synchronous expansions. Taking into consideration molecular clock studies [18, 36], it is tempting to hypothesize that TE bursts in the genomes of Erysiphaceae occurred independently of each other and might have preceded or followed adaptation to new hosts. Similar observations placing TE bursts around speciation times have been reported in the plant pathogen *Leptosphaeria maculans* [33, 61] and other eukaryotic organisms [62]. Theoretical models suggest that sudden TE expansions, when seen as a source of mutations, can push asexual organisms to a fitness optimum in adverse conditions [63, 64]. Given that at least powdery mildews of the genus *Blumeria* reproduce mainly clonally (asexual) as haploid organisms [12, 19] and their *formae speciales* exhibit narrow host specificity, our findings call for future studies to clarify the relationship between TE expansion and changes in the pathogen’s host range.

## Conclusions

We provide a greatly improved reference (isolate DH14) resource for the barley powdery mildew pathogen*,* and a near-continuous assembly of the highly divergent isolate RACE1. Gene order between these two isolates is retained at large scale, but locally disrupted. Using the new reference and supplementary transcriptomic and genomic data, we reassessed the secretome of grass powdery mildews and defined a core group of 190 SPs, which are likely to be indispensable for virulence. Inter-*formae speciales* comparisons further revealed that these virulence-related genes exhibit extensive CNV and sequence divergence, which reflects the phylogeny of these powdery mildews. *SP* genes are often locally clustered, but these clusters are evenly dispersed throughout the genome. TEs, which like the *SP* clusters are uniformly distributed in the *Bgh* genome and in part actively transcribed, experienced a recent lineage-specific expansion.

Taken together the results presented here indicate that *Bgh*, and more broadly the species *Blumeria graminis*, has a highly dynamic genome. While for other filamentous pathogens the existence of a “two-speed” genome has been suggested, the characteristics of the *Bgh* genome (even genome-wide distribution of TEs and *SPs*) indicate a “one/high-speed” genome for this pathogen and possibly its close relatives. It remains to be shown whether and how these features were enabled by the loss of genome defense modules (e.g. RIP), and if they contributed as springboard for the conquest of new host species (host jumps and host range expansions).

## Methods

### Genome sequencing

For DH14, genomic DNA was extracted as described in [11], while for RACE1 the protocol described in [65] was used. Subsequently, SMRTbell™ genomic libraries were generated and sequenced at the Earlham Institute (formally known as The Genome Analysis Centre, Norwich, United Kingdom) and at the Max Planck Genome Centre in Cologne (Germany) for DH14 and RACE1, respectively. The Pacific Biosciences (PacBio) RSII sequencing platform with either P5C3 (DH14) or P6C4 (RACE1) chemistry was deployed (Pacific Biosciences, Menlo Park, CA; [66]). A total of 21 SMRT cells achieved ~ 50× coverage for RACE1 (1,115,202 reads, 8357 bp average size), while for DH14 6 SMRT cells resulted in ~ 25× coverage (1,478,871 reads, 4540 bp average size). In addition, DH14 genomic DNA was sequenced at ~ 50× coverage with the Illumina MiSeq platform, providing 2 × 300 bp paired-end reads.

### Genome assembly

For both isolates the obtained PacBio reads were trimmed, corrected, and assembled using the Canu assembler (version 1.4; [20]) with default settings. The RACE1 assembly was further polished using Quiver (version 0.9.0; [67]) with default parameter settings. In the case of DH14 the resulting contigs were scaffolded with BESST (version 2.2.5; [68]) using previously published plasmid and fosmid libraries [11] and then polished using Illumina short reads and Pilon (version 1.18; [69]). To assess completeness of both assemblies we applied BUSCO (version 2.0.1; [70]) with default parameters searching against the Ascomycota database (ascomycota_odb9). To compare the assemblies with a previously published genetic map for *Bgh* [22], we obtained the nucleotide sequences of 80 single copy EST markers from this study and used BLASTN (BLAST+ version 2.3.0; [71]) to map these sequences against our genome assemblies (with e-value 1e^− 6^), thereby revealing their genomic location.

### RNA sequencing and alignment

For RACE1, we used RNA-seq data generated in the context of a previous study [9], and for DH14 we generated corresponding samples from barley leaf epidermal peels at 16 and 48 h after *Bgh* conidiospore inoculation for RNA-seq as described before [9]. The RNA-seq libraries were prepared by the Max Planck Genome Centre in Cologne (Germany) using the Illumina TruSeq stranded RNA sample preparation kit. The resulting libraries were subjected to paired-end sequencing (150 bp reads) using the Illumina HiSeq2500 Sequencing System.

To assess gene expression in DH14 and RACE1, the RNA-seq reads from both isolates were mapped to both genome assemblies under consideration of exon-intron structures using the splice aware aligner TopHat2 [72] with adjusted settings (−-read-mismatches 10 --read-gap-length 10 --read-edit-dist 20 --read-realign-edit-dist 0 --mate-inner-dist 260 --mate-std-dev 260 --min-anchor 5 --splice-mismatches 2 --min-intron-length 30 --max-intron-length 10,000 --max-insertion-length 20 --max-deletion-length 20 --num-threads 10 --max-multihits 10 --coverage-search --library-type fr-firststrand --segment-mismatches 3 --min-segment-intron 30 --max-segment-intron 10,000 --min-coverage-intron 30 --max-coverage-intron 10,000 --b2-very-sensitive) to account for sequence variability between isolates. To assess the expression of individual genes, we obtained raw fragment counts per gene from the mapped RNA-seq reads for both isolates (summarizing both time-points) using the featureCounts function (−t CDS -s 2 -M -p) of the Subread package (version 1.5.0-p1; [73]) and subsequently normalized these raw counts to fragment counts per kilobase CDS per million mapped reads (FPKM) for better comparability.

Expression of TEs in the isolate DH14 was assessed by mapping pooled RNA-seq reads coming from the 16 and 48 hpi DH14 samples with STAR [74], using the RepeatMasker-derived gff file as annotation. Raw counts per TE annotation were obtained using the --quantMode GeneCounts option.

### Gene annotation

The prediction of DH14 and RACE1 gene models was performed using the MAKER pipeline (version 2.28; [75]), which integrates different ab initio gene prediction tools together with evidence from EST and protein alignments.

For DH14, initially the previous gene models (v3, https://www.ebi.ac.uk/ena/data/view/GCA_000151065.3) were transferred to the new assembly as described [28]. Then an additional round of annotation followed, incorporating ESTs assembled from public *Bgh* datasets (Additional file 1: Table S13) using Trinity [76], protein datasets (Additional file 1: Table S13), as well as trained prediction models for AUGUSTUS [77], SNAP [78] and GeneMark-ES [79] as supporting evidence. All the annotations were subsequently manually curated using Web Apollo [80], removing unsupported gene models.

For RACE1, we performed a complete de novo annotation, as there were no previous gene models available. For this purpose, the MAKER pipeline was first run using AUGUSTUS [77] with species model *Botrytis cinerea* and GeneMark-ES [79] for ab initio gene prediction together with transcript and protein alignment evidence. The corresponding alignment evidence was created from BLAST and Exonerate [81] alignments of the DH14 protein sequences as well as RACE1 protein and transcript sequences. The RACE1 transcript and protein sequences for these alignments were obtained from the corresponding RNA-seq data via a transcriptome de novo assembly using Trinity [76] with default parameter settings for paired-end reads and subsequent open reading frame/peptide prediction using TransDecoder [76] with default settings. The resulting gene models from the first MAKER run were used as initial training set for another ab initio prediction tool, SNAP [78]. Next, the annotation pipeline was re-run including all three ab initio prediction tools together with the transcript and protein alignment evidence, thus generating a second, improved training set for SNAP. After re-training SNAP on this set, the complete annotation pipeline was run a third time to yield the final RACE1 gene models. For both isolates, the obtained gene models were manually curated using Web Apollo [80], to correct for errors and remove poorly supported gene models. The mitochondrial genome of DH14 was annotated using RNAweasel and MFannot (http://megasun.bch.umontreal.ca/RNAweasel/).

### Identification of orthologous genes and gene groups

Groups of orthologous genes (orthogroups) were inferred from DH14 and RACE1 using OrthoFinder (version 1.1.8; [82]) with the inflation value I set to 1.2. To further resolve ambiguities in the orthogroups and detect additional relationships between more dissimilar sequences, subsequently, a manual screening of gene positions and co-linearity in the two genomes was performed and the ortholog assignment was refined accordingly.

Isolate-specific genes were identified by combining the results of the OrthoFinder analysis with the alignment results for the RNA-seq data from both isolates. Explicitly, a gene was only considered to be specific for one isolate if, after OrthoFinder analysis and manual refinement, there was no orthologous gene detectable in the genome of the other isolate and additionally also no RNA-seq fragment (read pair) from the other isolate were detected to map against this gene (raw count ≤1). The fragment count per gene was calculated from the mapped RNA-seq read pairs (with mapping quality > 0) using featureCounts (version 1.5.0; [73]) with adjusted settings (−s 2 -p -M), based on the curated gene models. For identification of isolate-specific gene expression the raw fragment counts were further normalized to FPKM values, to adjust for potential differences in coding sequence length and RNA-seq read depth between isolates.

To calculate non-synonymous (dN) and synonymous (dS) substitution rates between DH14 and RACE1, we first aligned the protein sequences for each of the manually curated orthologous gene pairs with ClustalW (version 2.1; [83]). Subsequently, the protein alignments were converted to codon alignments using PAL2NAL (version 14; [84]) and dN and dS rates were estimated from these codon alignments using the yn00 function of the PAML package (version 4.4; [85]).

### Whole-genome comparison

A whole-genome alignment between DH14 and RACE1 was generated using the nucmer and dnadiff functions of the MUMmer software (version 3.9.4; [31]) with default settings. Alignment gaps (≥1 kb) and inverted regions (≥10 kb) were extracted from the dnadiff 1coords output file. To construct circular visualizations of this alignment, we used the Circos software (version 0.62.1; [86]). For the overview plot, we initially picked the 8 and 11 largest contigs from the DH14 and RACE1 genomes, based on the corresponding N50 values. For each of these contigs we then extracted any further aligning contigs from the other isolate, for which the sum of all aligned regions (with size ≥2 kb and sequence similarity ≥75%) covered at least 10% of both contigs. For the more detailed view of the large-scale rearrangements, we initially selected the contigs with the observed breakpoints and extracted all aligning contigs from the other isolate, for which the sum of all aligning regions (with size ≥1 kb and sequence similarity ≥75%) covered at least 25% of at least one of the contigs. The circular visualizations also depict gene and TE densities along the genome, which were calculated in 10 kb sliding windows (moving by 1 kb at each step) as fraction of bp within each window that is covered by a gene annotation or TE, respectively. For the linear alignment visualizations of the two largest DH14 contigs, we included all aligning regions of at least 1 kb and plotted the corresponding sequence identities from the MUMmer output together with the corresponding gene and TE densities along those contigs. The detailed view of the local inversions observed within the otherwise syntenic alignment between RACE1 tig00005299 and DH14 scaffold 35 was generated with SyMap (version 4.2; [87]).

### Secretome and core effectorome analysis

The secretomes of all genomes assayed here were identified based on the presence of a signal peptide as detected with SignalP (version 4.1; [88]) and absence of any transmembrane domain in the mature protein as predicted by TMHMM (version 2.0; [89]). Functional domain annotation of the proteomes was performed with InterProScan [90].

To define the core *Blumeria*-specific effectorome, we assembled the genomes of the *formae speciales avenae, dicocci, dactylis, lolii, poae, secalis* and *triticale* using the publicly available raw Illumina reads for the isolates AVE, LIB1609, DAC, LOL, POAE, S1459 and T1–20 (Additional file 1: Table S13). The assemblies were carried out using ABySS 2.0.2 [91], and the gene space coverage with BUSCO (Additional file 1: Table S14). For the *forma specialis tritici* the reference assembly of the isolate 96,224 was used. The resulting contig sequences were de novo-annotated with one round of MAKER using the same settings as for the DH14 annotation (described previously, also https://github.com/lambros-f/blumeria_2017).

To remove widely conserved, non-*Blumeria* specific proteins, all predicted secreted proteins were used as query in BLASTP searches (version 2.5.0+) against the NCBI non-redundant protein database (nr) with the e-value threshold of 10e-5. Additionally, to derive the presence of core *Blumeria*-specific SPs, an ortholog search was performed using OrthoFinder and the predicted proteomes of the *formae speciales*. To remove potential bias originating from possible conserved secretion signal peptide sequences, the predicted *Bgh* SPs were inserted in the analysis as mature peptides.

To generate a maximum likelihood-based phylogenetic tree for the SPs, all the *Bgh* DH14 mature peptide sequences were aligned with MAFFT v7.310 (−-maxiterate 1000 –localpair; [92]). Afterwards IQ-TREE multicore version 1.6.beta4 [93] and ModelFinder [94] were used to select an optimum substitution model and generate the final ML tree. The substitution model used was VT + R8.

To further assess whether *SPs* or *CSEPs* are located in gene sparse regions, BEDTools [95] with the functions complement and closest was utilized to calculate the 5′ and 3′ intergenic space lengths for all genes. The resulting tables were introduced in to R in order to generate the corresponding figure using ggplots2. The corresponding R script is deposited in https://github.com/lambros-f/blumeria_2017. As further control we extracted a set of ascomycete core ortholog genes (*COGs*) based on the BUSCO Ascomycota odb9 hidden Markov models (http://busco.ezlab.org/datasets/ascomycota_odb9.tar.gz).

### Divergence landscapes of transposable elements

To generate divergence landscapes for the TEs of the *Letiomycete* fungi, repeat elements were identified in all genomes using RepeatMasker (version 4.0.7, http://www.repeatmasker.org/) with default parameters and *fungi* as the query species based on the Repbase database version 20,150,807 (downloaded on 2016/06/09). Afterwards the RepeatMasker align output (.aln) was parsed using previously described Perl scripts (https://github.com/4ureliek/Parsing-RepeatMasker-Outputs, [96]). The selection of genomes used for this analysis (Additional file 1: Table S13) and their relation to *Bgh* was derived from the orthology analysis of their proteomes using OrthoFinder [82]. For the analysis of the dicot-infecting powdery mildews the publicly available assemblies were used (Additional file 1: Table S13), or in the case of *G. orontii* isolate MGH1 the PacBio reads were assembled with Canu as described previously. It should be noted that proportions of TE types differ in part from previous publications due to usage of the public Repbase database in this work and customized TE libraries in [11, 97].

### Duplicate gene search and copy number variation analysis

In order to assess whether duplicate genes exist in the *Bgh* DH14 and RACE1 genomes, MCScanX was used [32] with the default parameters. Subsequent analysis to derive copy number variation in all *formae speciales* and their corresponding isolates was carried out as follows. All genomic reads were first quality trimmed using Trimmomatic (version 0.36; [98]) and then aligned to the DH14 genome using BWA-MEM [99]. The resulting bam file was sorted using Picard (http://broadinstitute.github.io/picard) and the read depth per bp was extracted using BEDTools [95]. The copy number of each SP was calculated by the average per bp coverage of the gene model by the respective mapped reads, divided by the average coverage of all 805 SPs using custom R scripts. The distance matrix was computed using the Euclidean method, and the heatmap was generated using heatmap.2 from the package gplots. The bash and R scripts used for this analysis can be found in https://github.com/lambros-f/blumeria_2017.

### Phylogeny of the isolates

The phylogenetic relationship of the *formae speciales* and their corresponding isolates was derived from SNPs. The genomic reads of every isolate were mapped to the DH14 reference genome with BWA-MEM [100], and the GATK best practices pipeline [101, 102] was used for SNP discovery, as previously described [103]. Afterwards, VCFtools 0.1.15 [104] was deployed with the option --max-missing 1 to keep only common SNPs, resulting in 1,070,264 sites. The resulting VCF files were parsed with custom Perl and bash scripts (https://github.com/lambros-f/blumeria_2017) and imported to SplitsTree [105] to generate a cladogram based on an UPGMA tree.

### SNP analysis

For isolates A6 and K1, SNPs to DH14 were identified with GATK [101, 102] from the BWA-MEM [100] alignment of short sequence reads as described above. For RACE1, SNPs to DH14 were identified using the nucmer and dnadiff functions of the MUMmer software (version 3.9.4; [31]) with default settings. Subsequently, for all three isolates, we calculated the SNP frequency as a function of the genomic location by using a 10 kb sliding window that moved 1 kb at each step for all DH14 contigs larger than 50 kb. To further examine the distribution of low and high SNP frequencies, we applied the expectation-maximization (EM) algorithm (function normalmixEM, R-package mixtools) to fit a two-component mixture model to the observed SNP frequencies as described previously [12, 19].

## Additional files

Additional file 1: **Table S1.** BUSCO genome completeness analysis. **Table S2.** Scaffolds with telomeric repeats at their ends in the DH14 and RACE1 assembly. **Table S3.** Assosiation table for old and new *Bgh* gene model IDs. **Table S4.** Manually currated orthology relationships between RACE1 and DH14. **Table S5.** Isolate-specific genes in DH14 and RACE1. **Table S6.** Isolate-specific gene expression in DH14 and RACE1. **Table S7.** Clustering of secreted protein coding genes in the *Bgh* DH14 genome. **Table S8.** MCScanX analysis for the identification of duplicate genes. **Table S9.** Functional domains of dispersed duplicate genes in *Sclerotinia sclerotiorum*. **Table S10.** Secreted/Non-secreted duplications in the *Bgh* DH14 genome. **Table S12.** Raw read count for the top 20 expressing transposable elements in pooled 16 h & 48 h dpi DH14 RNA-seq dataset. **Table S13.** All datasets used for analyses in this study. **Table S14.** BUSCO analysis for the *forma specialis* genomes used for orthology calling between the CSEPs. (XLSX 1151 kb)

Additional file 2: **Figure S1.** Comparative alignment of the *Bgh* DH14 and RACE1 genome assemblies with a *Bgh* genetic map. The distribution and ordering of 80 single copy EST markers across 30 linkage groups of a previously published genetic map [22] is visualized in relation to the corresponding genomic locations of these markers in the DH14/RACE1 assemblies. Each box represents a specific genomic contig or linkage group (LG), respectively, and the numbers inside the boxes specify the marker positions on the corresponding contig (in bp) or linkage group (in cM). The corresponding marker identifiers are given next to the boxes. Dashed connector lines represent markers for which the genomic location and genetic map are consistent. Discrepancies between assembly and genetic map are indicated by solid connectors, with black lines representing markers whose location is consistent between assemblies but different from the genetic map, and colored lines representing markers with differences to the genetic map that are specific to either DH14 (dark pink) or RACE1 (blue). (PDF 4159 kb)

Additional file 3: ** Figure S2.** Involvement of TEs in chromosomal organization. (A) Density of different categories of repetitive elements and genes per 50 kb sliding windows in selected scaffolds with putative centromeric regions. A subset of *Tad1*-like LINE elements that are associated with putative centromeric regions are highlighted in green. (B) Box plots of the 5′ and 3′ intergenic distances for ascomycete core ortholog genes (*COGs*), *CSEPs* and other secreted protein-coding genes that do not fulfil the *CSEP* criteria (“other *SPs*”). No statistically significant differences were detected for the 5′ distances (*p* = 0.382; ANOVA) and differing letters indicate statistically significant differences between groups for the 3′ distances (*p* < 0.05; ANOVA with Tukey post hoc tests). (C) Plots depicting by color-code the dN/dS ratio of each gene of the three different groups (*COGs*, *CSEPs*, other *SPs*) in relation to their flanking intergenic length. Genes with dS values of 0 are not shown. (PDF 1888 kb)

Additional file 4: ** Figure S3.** Mitochondrial genomes of *Bgh*. (A) Map and corresponding annotation of the mitochondrial genome of *Bgh* isolate DH14 resulting from an RNAweasel and MFannot run. (B) Nucleotide sequence alignment between the DH14 (x-axis) and RACE1 (y-axis) mtDNA using NUCmer, indicating a putative partial duplication in RACE1. (PDF 224 kb)

Additional file 5: ** Figure S4.** Comparative visualization of the genomic loci harboring *AVR*_*a1*_ and *AVR*_*a13*_ in the *Bgh* isolates DH14 and RACE1. (A) Organization of the genomic locus harboring the previously identified *AVR*_*a1*_ (orange arrows) and some of its flanking genes in DH14 and RACE1. (B) Organization of the genomic locus harboring the previously identified *AVR*_*a13*_ (green arrows) and some of its flanking genes in DH14 and RACE1. (PDF 1206 kb)

Additional file 6: **Figure S5.** Variation in the mating type locus in the *Bgh* isolates DH14 and RACE1. Organization of the genomic loci containing the mating type genes (*MAT-1-1-1*, *MAT-1-1-3* and *MAT-1-2-1*) and some of its flanking genes. As DH14 and RACE1 are of opposite mating types, the structure of the mating type locus differs between the two isolates. The genomic locus in RACE1, which is of the MAT-1-1 mating type, was assembled completely, while the respective locus in DH14 (MAT-1-2 mating type) is distributed on two scaffolds. (PDF 1139 kb)

Additional file7: ** Figure S6.** Evidence for two large-scale genomic rearrangements between the isolates DH14 and RACE1. Circos diagram showing evidence for large-scale genomic rearrangements between DH14 and RACE1. The two scaffolds/contigs in the assemblies of DH14 and RACE1 with internal alignment breaks and the corresponding aligning scaffolds/contigs in the other isolate were extracted for visualization. Syntenic regions and alignment breaks were identified based on a whole-genome alignment, and aligning regions of at least 1 kb between the two isolates (with nucleotide sequence similarity ≥75%) are connected with lines in the circular plot. Lines within the syntenic blocks directly flanking the breaks are shown in color while lines in all other blocks are depicted in grey. The positions of the observed alignment breaks are marked by arrowheads colored in green (three breaks likely involved in the same event) and brown (two breaks likely involved in the same event). The surrounding circles represent from the outside: on the right side the DH14 scaffolds (pink) and on the left side the RACE1 (blue), with all unaligned regions (≥ 0.5 kb) indicated as white gaps on the scaffolds/contigs; the gene density (green) and TE density (blue) calculated in 10 kb sliding windows; the locations of all genes predicted to code for SPs; the locations of isolate-specific genes coding for SPs (dark red) or any other proteins (black); and isolate-specific additional gene copies/paralogs coding for SPs (pink) or any other proteins (grey). (PDF 1023 kb)

Additional file 8: ** Figure S7.** Frequency of single-nucleotide polymorphisms (SNPs) between *Bgh* isolates. (A) Kernel density plot of the SNP frequencies per kb in 10 kb sliding windows, observed for the three *Bgh* isolates A6, K1 and RACE1 relative to the reference isolate DH14. The plot depicts Gaussian kernel density estimates calculated at a smoothing bandwidth of 0.12. (B) Average SNP frequencies for A6, K1 and RACE1 in 10 kb sliding windows of low and high SNP density as estimated by a two-component mixture model that was fitted to the observed SNP frequencies using the expectation-maximization algorithm. Error bars indicate the corresponding standard deviations estimated by the mixture model. (PDF 294 kb)

Additional file 9: ** Figure S8.** Distribution of SP and non-SP coding genes in *Bgh* DH14 scaffolds larger than 1 MB. (A) Density plots of SP coding genes (orange), non-SP coding genes (purple) and different types of TE elements (gray) in 50 kb sliding windows. Scaffolds depicted here were selected based on their size (> 1 MB) and represent ~ 87% of the total genomic sequence. (B) Number of SP coding genes per scaffold plotted against the respective total scaffold size, showing positive correlation (*r* = 0.88, *p* < 0.001). (PDF 4282 kb)

Additional file 10: ** Figure S9.** CNV of widely conserved genes between *B. graminis formae speciales*. Heatmap illustrating the copy number of genes with putatively widely conserved functions. Using the same pipeline as for the generation of Fig. 3a, all 34 genes with a PFAM annotation including the terms “tubulin” (highlighted in red) or “actin” (highlighted in green) and 49 genes coding for non-SP genes with conserved domains were used as a control dataset to estimate the error rate of the CNV calling pipeline. The heatmap depicts the color-coded copy number of these genes per individual genome of various *B. graminis formae speciales* (*avenae*, *dactylis*, *dicocci*, *hordei, lolii*, *poae, secalis*, *triticale* and *tritici*), each represented by one or more isolates as indicated on the right. The dendrogram on the left is based on the hierarchical clustering (Euclidean method) of the CNV values for every dataset. (PDF 466 kb)

Additional file 11: ** Figure S10.** Secretome orthology relations and core effectorome phylogeny. (A) Heatmap of *SP* orthologs found for the *formae speciales* genomes after ortholog clustering using OrthoFinder on the predicted proteomes of the isolates T1–20, S1459, LIB1609, DAC, 96224, LOL, AVE, POAE, DH14. Every column corresponds to one of the 805 *Bgh* DH14 predicted *SPs*, while color-coding depicts the number of orthologs in the corresponding orthogroup. Hierarchical clustering (Euclidean method) for the *formae speciales* and the *SPs* are given on the left and the top of the heatmap, respectively. (B) Maximum likelihood phylogeny tree of the 805 SPs. The tree was generated using IQ-TREE based on the mature peptide sequences of the *Bgh* DH14 SPs. Orange edge tips indicate the 190 core CSEPs which have orthologs in all *formae speciales*. The scale bar indicates the number of amino-acid substitutions per site. (PDF 2195 kb)

Additional file 12: ** Figure S11.** Representatives of the genus *Blumeria* show less TE divergence than representatives of the genera *Erishyphe* and *Golovinomyces*. (A) The histograms indicate the frequency of a given sequence divergence for TE families of 10 *B. graminis* genomes. The genomes, which were assembled based on various sequencing platforms (PacBio or Illumina), were surveyed for their repeat content and repeat landscapes for each genome based on % nucleotide divergence to the consensus TE sequences were calculated out of the RepeatMasker output using Perl scripts. Sequence divergence (x-axis) is plotted against frequency (number of sequences; y-axis) for each of the genomes. (B) The histograms indicate the frequency of a given sequence divergence for TE families of 3 dicot-infecting powdery mildew species (*Erysiphe pisi*, *E. necator* and *Golovinomyces orontii*). The genomes, which were assembled based on various sequencing platforms (PacBio, ABI Solid or Illumina), were surveyed for their repeat content and repeat landscapes for each genome based on % nucleotide divergence to the consensus TE sequences were calculated out of the RepeatMasker output using Perl scripts. Sequence divergence (x-axis) is plotted against frequency (number of sequences; y-axis) for each of the genomes. (PDF 255 kb)

## Abbreviations

*AVR*: Avirulence effector gene
*Bgh*: *Blumeria graminis* f.sp. *hordei*
CNV: Copy number variation
CSEP: Candidate secreted effector protein
EST: Expressed sequence tag
FPKM: Fragments per kilobase [sequence length] and million [sequenced fragments]
mtDNA: mitochondrial DNA
SNP: Single nucleotide polymorphism
SP: Secreted protein
TE: Transposable element
