# Supplementary figures and images for: Signatures of host specialization and a recent transposable element burst in the dynamic one-speed genome of the fungal barley powdery mildew pathogen
Source: BMC Genomics. 2018 May 22;19:381. doi: 10.1186/s12864-018-4750-6 (PMC5964911; doi:10.1186/s12864-018-4750-6)

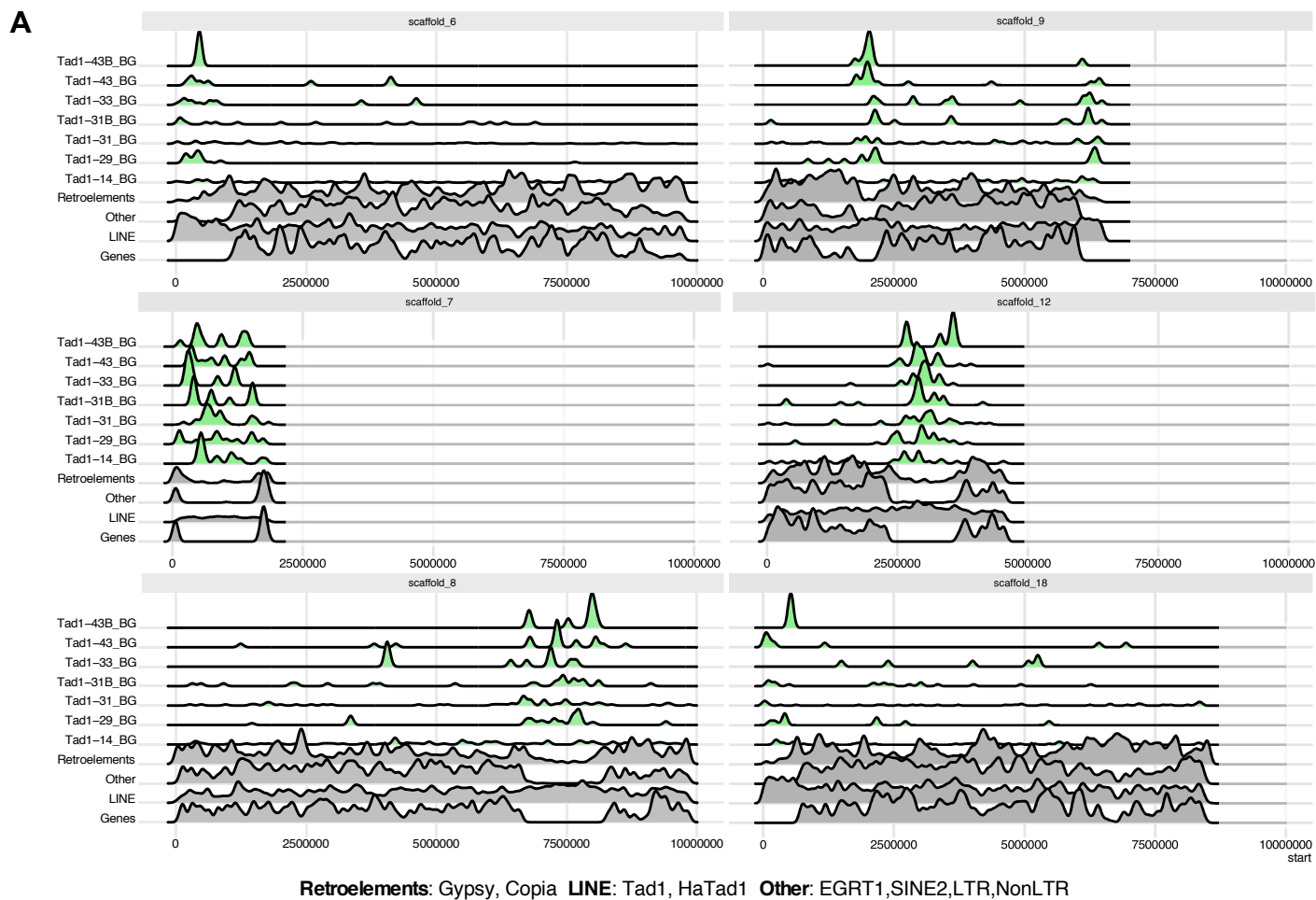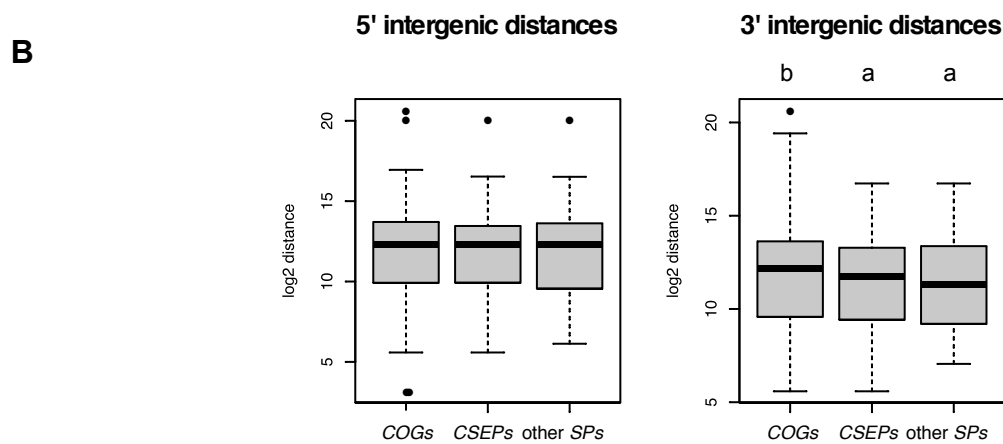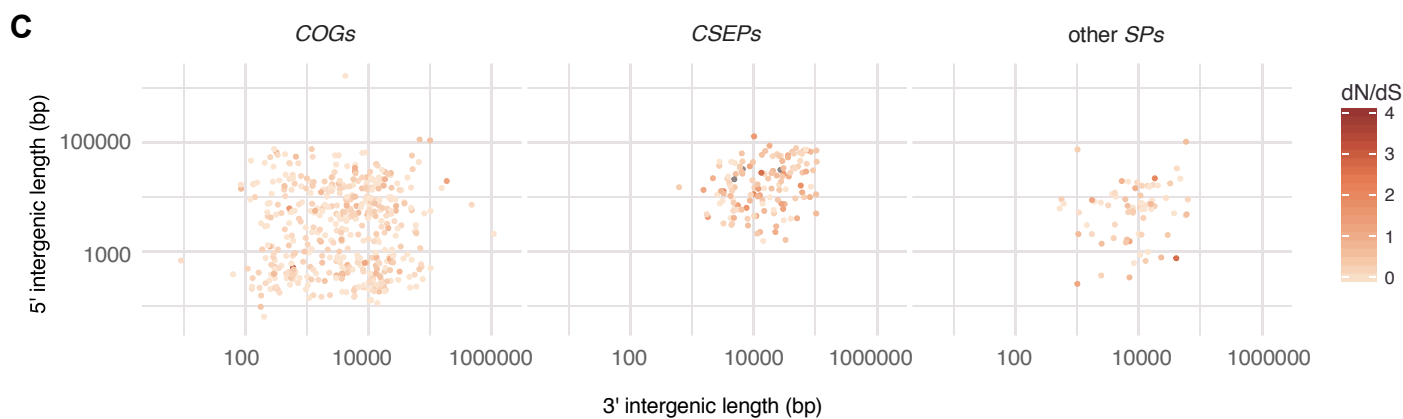

Supplement: Supplementary file 3 — Figure S2. Involvement of TEs in chromosomal organization. (A) Density of different categories of repetitive elements and genes per 50 kb sliding windows in selected scaffolds with putative centromeric regions. A subset of Tad1-like LINE elements that are associated with putative centromeric regions are highlighted in green. (B) Box plots of the 5′ and 3′ intergenic distances for ascomycete core ortholog genes (COGs), CSEPs and other secreted protein-coding genes that do not fulfil the CSEP criteria (“other SPs”). No statistically significant differences were detected for the 5′ distances (p = 0.382; ANOVA) and differing letters indicate statistically significant differences between groups for the 3′ distances (p < 0.05; ANOVA with Tukey post hoc tests). (C) Plots depicting by color-code the dN/dS ratio of each gene of the three different groups (COGs, CSEPs, other SPs) in relation to their flanking intergenic length. Genes with dS values of 0 are not shown. (PDF 1888 kb) [file 12864_2018_4750_MOESM3_ESM.pdf]

**A**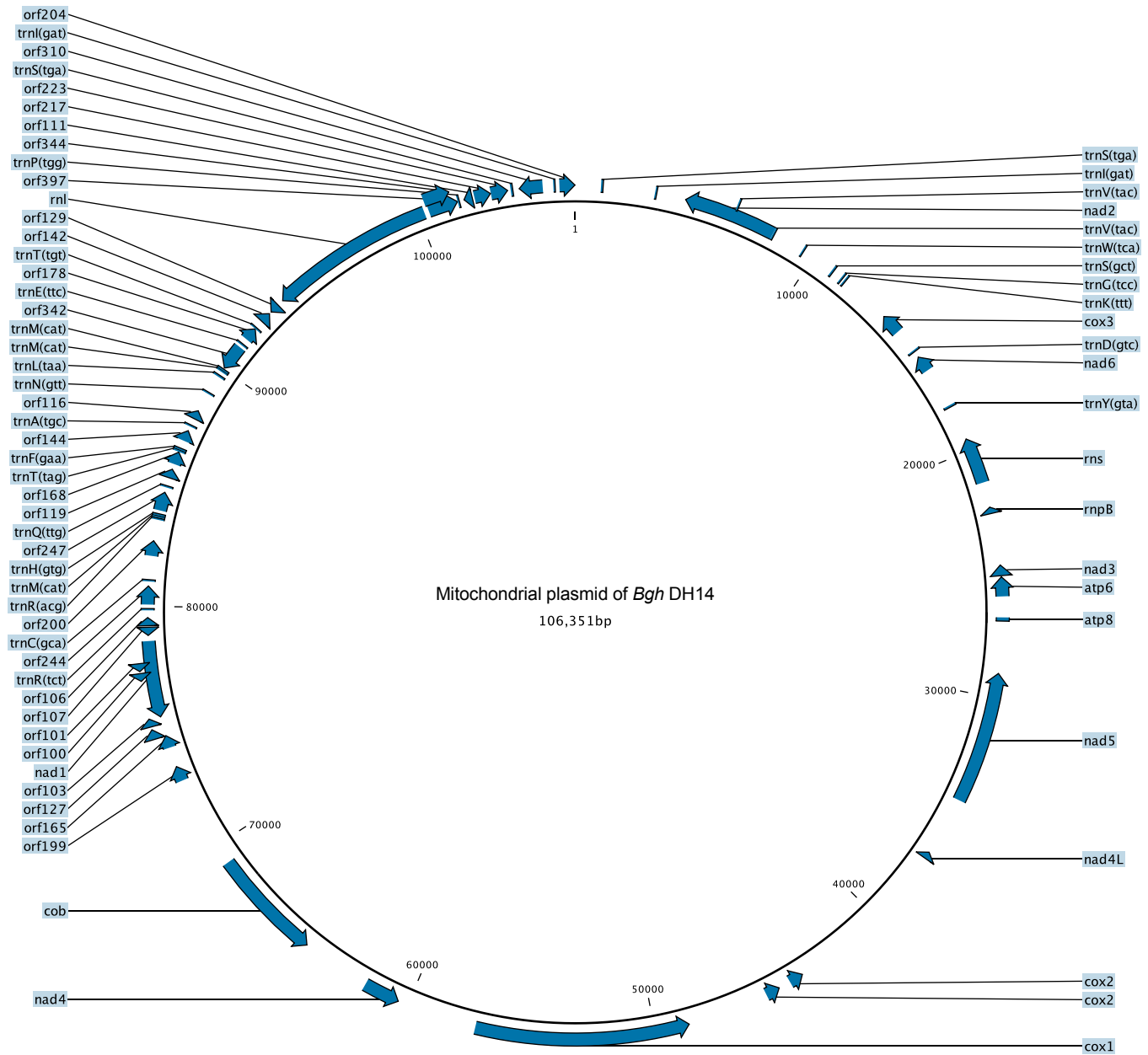**B**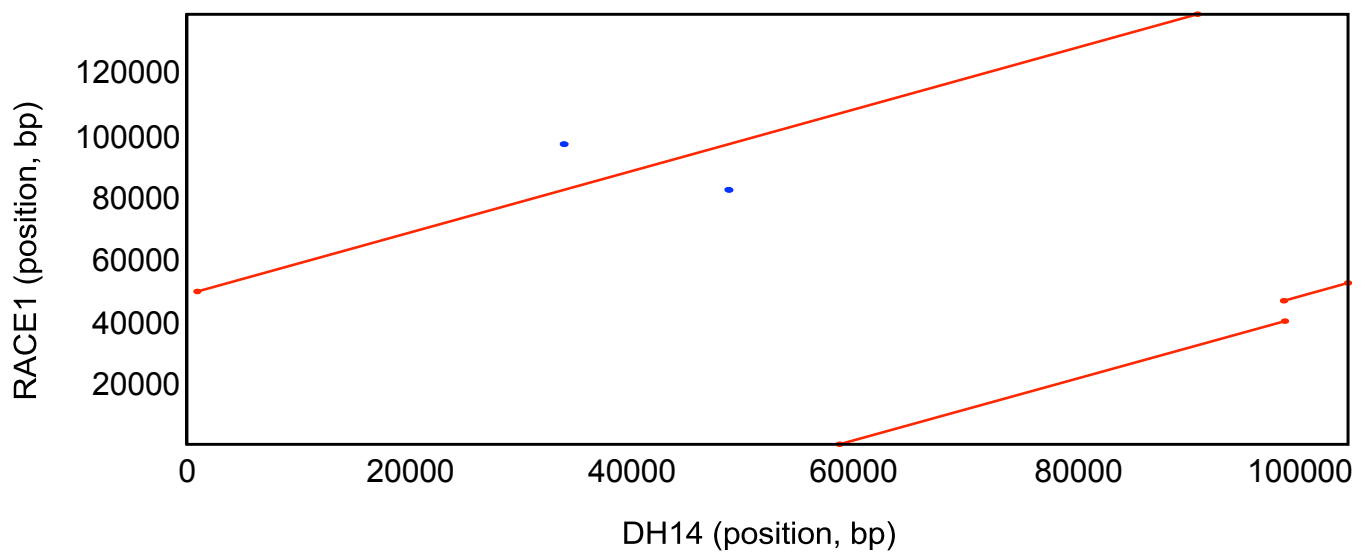

Supplement: Supplementary file 4 — Figure S3. Mitochondrial genomes of Bgh. (A) Map and corresponding annotation of the mitochondrial genome of Bgh isolate DH14 resulting from an RNAweasel and MFannot run. (B) Nucleotide sequence alignment between the DH14 (x-axis) and RACE1 (y-axis) mtDNA using NUCmer, indicating a putative partial duplication in RACE1. (PDF 224 kb) [file 12864_2018_4750_MOESM4_ESM.pdf]

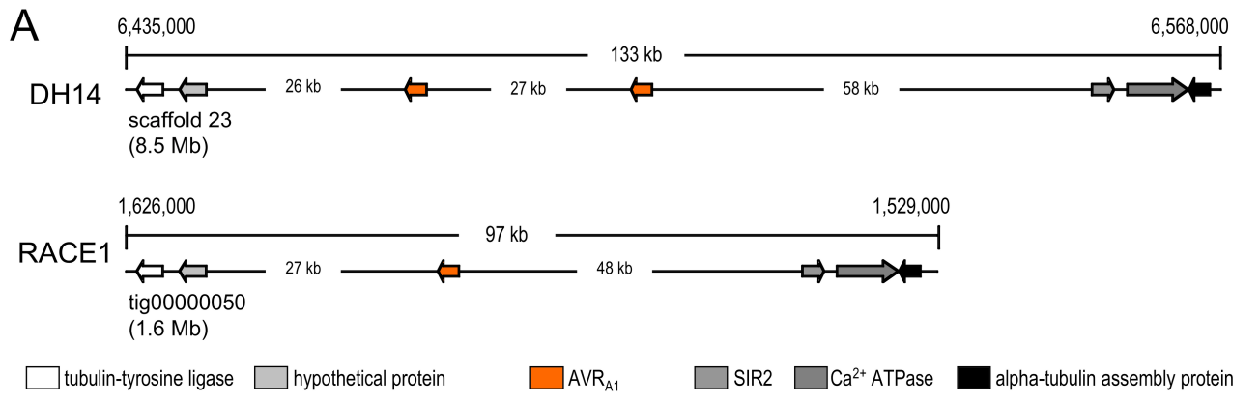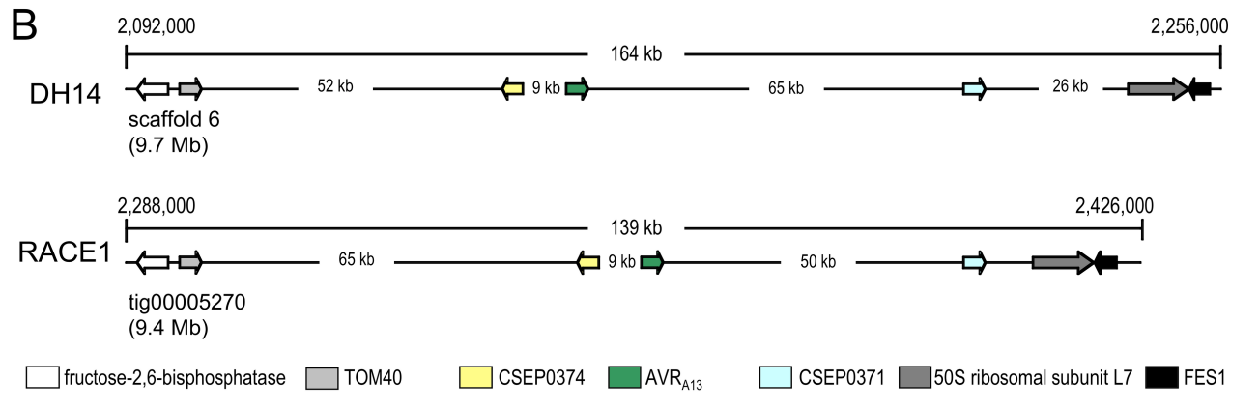

Supplement: Supplementary file 5 — Figure S4. Comparative visualization of the genomic loci harboring AVRa1 and AVRa13 in the Bgh isolates DH14 and RACE1. (A) Organization of the genomic locus harboring the previously identified AVRa1 (orange arrows) and some of its flanking genes in DH14 and RACE1. (B) Organization of the genomic locus harboring the previously identified AVRa13 (green arrows) and some of its flanking genes in DH14 and RACE1. (PDF 1206 kb) [file 12864_2018_4750_MOESM5_ESM.pdf]

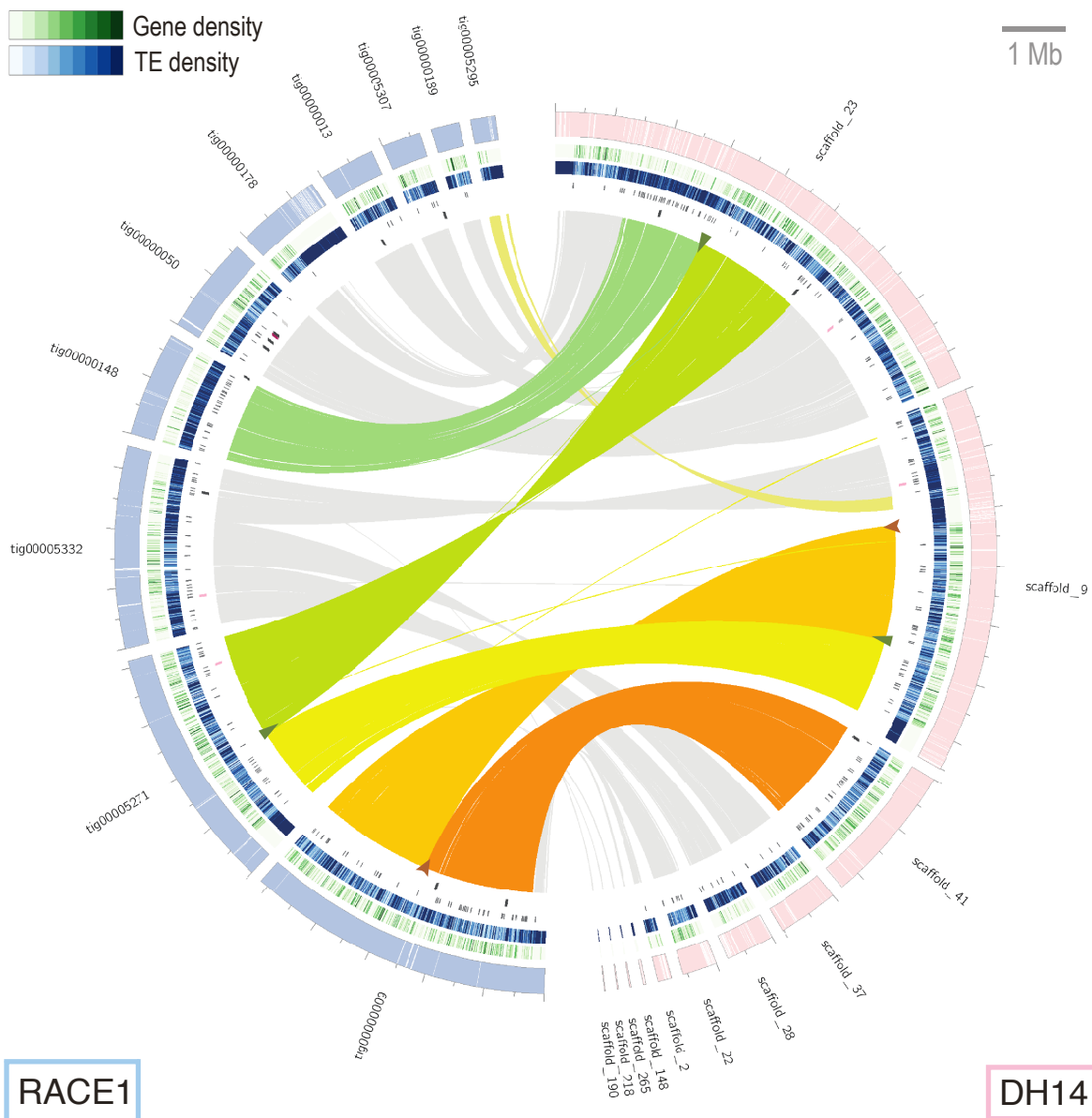

Supplement: Supplementary file 7 — Figure S6. Evidence for two large-scale genomic rearrangements between the isolates DH14 and RACE1. Circos diagram showing evidence for large-scale genomic rearrangements between DH14 and RACE1. The two scaffolds/contigs in the assemblies of DH14 and RACE1 with internal alignment breaks and the corresponding aligning scaffolds/contigs in the other isolate were extracted for visualization. Syntenic regions and alignment breaks were identified based on a whole-genome alignment, and aligning regions of at least 1 kb between the two isolates (with nucleotide sequence similarity ≥75%) are connected with lines in the circular plot. Lines within the syntenic blocks directly flanking the breaks are shown in color while lines in all other blocks are depicted in grey. The positions of the observed alignment breaks are marked by arrowheads colored in green (three breaks likely involved in the same event) and brown (two breaks likely involved in the same event). The surrounding circles represent from the outside: on the right side the DH14 scaffolds (pink) and on the left side the RACE1 (blue), with all unaligned regions (≥ 0.5 kb) indicated as white gaps on the scaffolds/contigs; the gene density (green) and TE density (blue) calculated in 10 kb sliding windows; the locations of all genes predicted to code for SPs; the locations of isolate-specific genes coding for SPs (dark red) or any other proteins (black); and isolate-specific additional gene copies/paralogs coding for SPs (pink) or any other proteins (grey). (PDF 1023 kb) [file 12864_2018_4750_MOESM7_ESM.pdf]

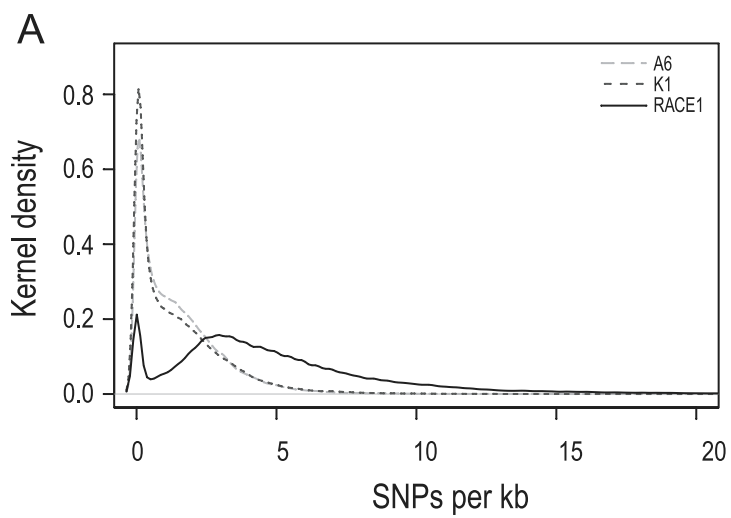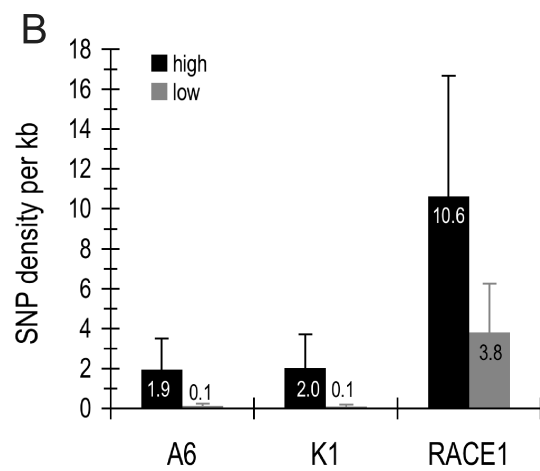

Supplement: Supplementary file 8 — Figure S7. Frequency of single-nucleotide polymorphisms (SNPs) between Bgh isolates. (A) Kernel density plot of the SNP frequencies per kb in 10 kb sliding windows, observed for the three Bgh isolates A6, K1 and RACE1 relative to the reference isolate DH14. The plot depicts Gaussian kernel density estimates calculated at a smoothing bandwidth of 0.12. (B) Average SNP frequencies for A6, K1 and RACE1 in 10 kb sliding windows of low and high SNP density as estimated by a two-component mixture model that was fitted to the observed SNP frequencies using the expectation-maximization algorithm. Error bars indicate the corresponding standard deviations estimated by the mixture model. (PDF 294 kb) [file 12864_2018_4750_MOESM8_ESM.pdf]

**A**

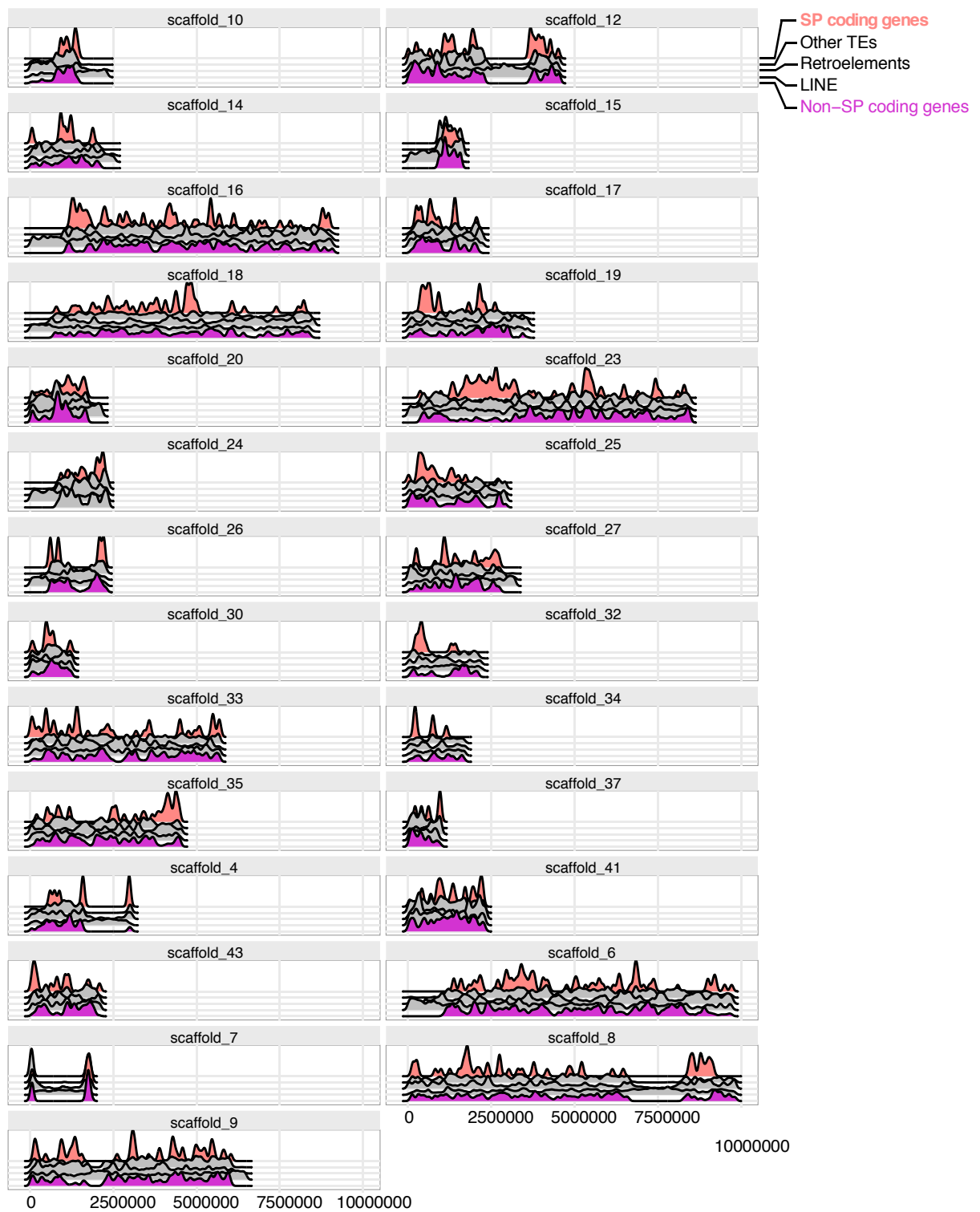

**B**

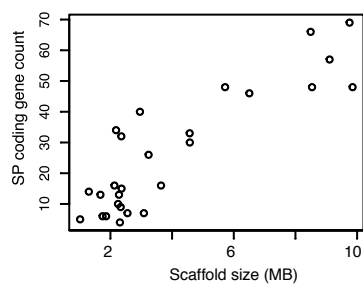

Supplement: Supplementary file 9 — Figure S8. Distribution of SP and non-SP coding genes in Bgh DH14 scaffolds larger than 1 MB. (A) Density plots of SP coding genes (orange), non-SP coding genes (purple) and different types of TE elements (gray) in 50 kb sliding windows. Scaffolds depicted here were selected based on their size (> 1 MB) and represent ~ 87% of the total genomic sequence. (B) Number of SP coding genes per scaffold plotted against the respective total scaffold size, showing positive correlation (r = 0.88, p < 0.001). (PDF 4282 kb) [file 12864_2018_4750_MOESM9_ESM.pdf]

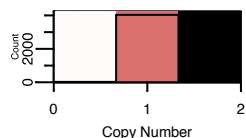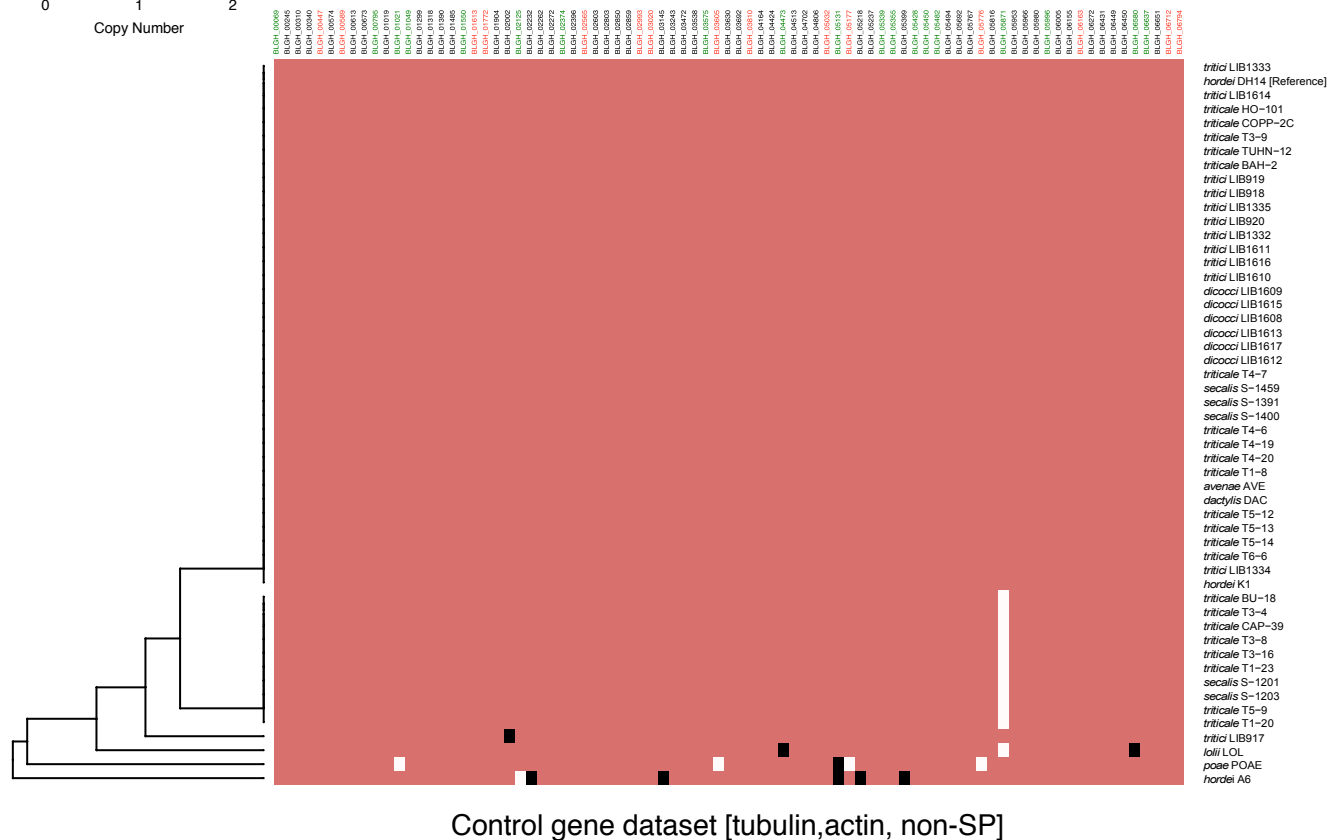

Supplement: Supplementary file 10 — Figure S9. CNV of widely conserved genes between B. graminis formae speciales. Heatmap illustrating the copy number of genes with putatively widely conserved functions. Using the same pipeline as for the generation of Fig. 3a, all 34 genes with a PFAM annotation including the terms “tubulin” (highlighted in red) or “actin” (highlighted in green) and 49 genes coding for non-SP genes with conserved domains were used as a control dataset to estimate the error rate of the CNV calling pipeline. The heatmap depicts the color-coded copy number of these genes per individual genome of various B. graminis formae speciales (avenae, dactylis, dicocci, hordei, lolii, poae, secalis, triticale and tritici), each represented by one or more isolates as indicated on the right. The dendrogram on the left is based on the hierarchical clustering (Euclidean method) of the CNV values for every dataset. (PDF 466 kb) [file 12864_2018_4750_MOESM10_ESM.pdf]

**A**

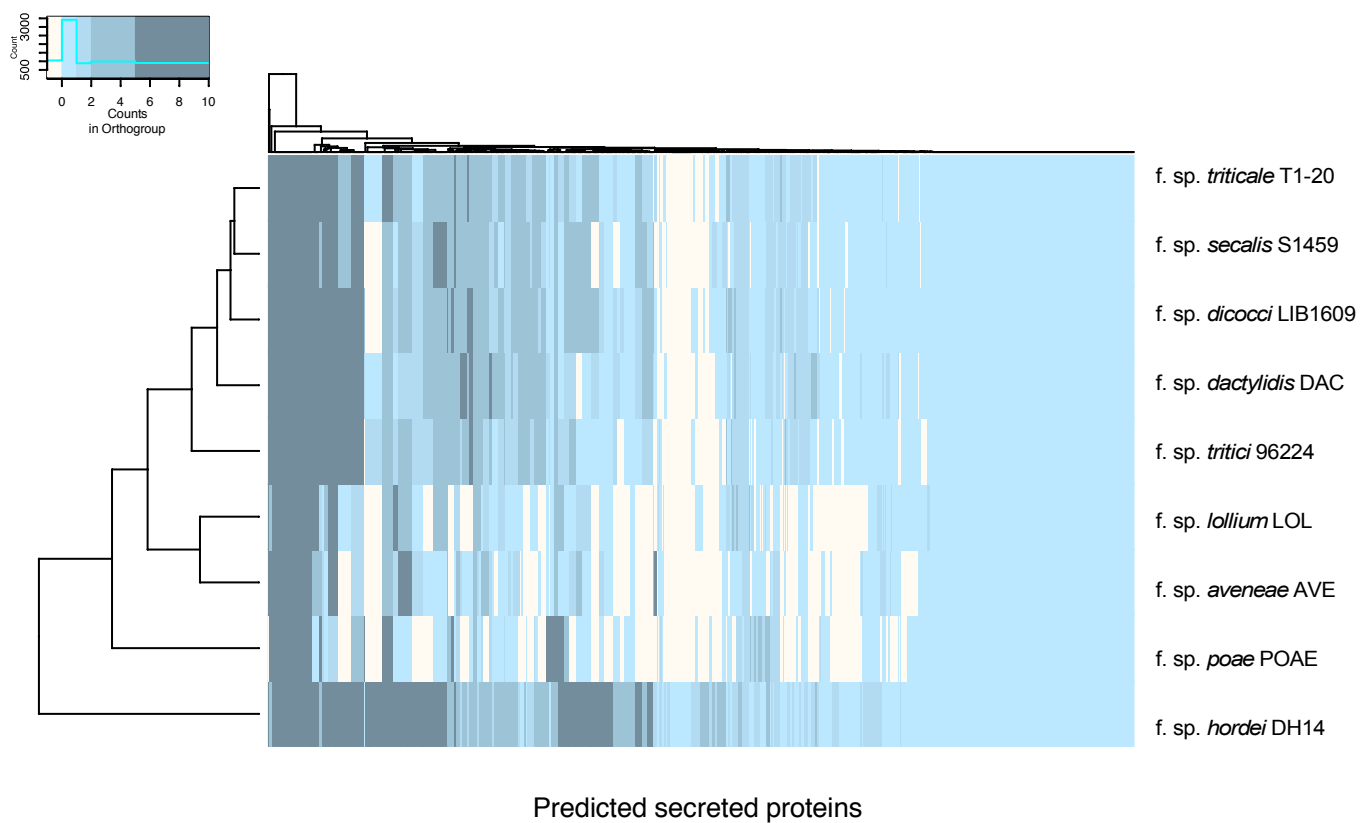

**B**

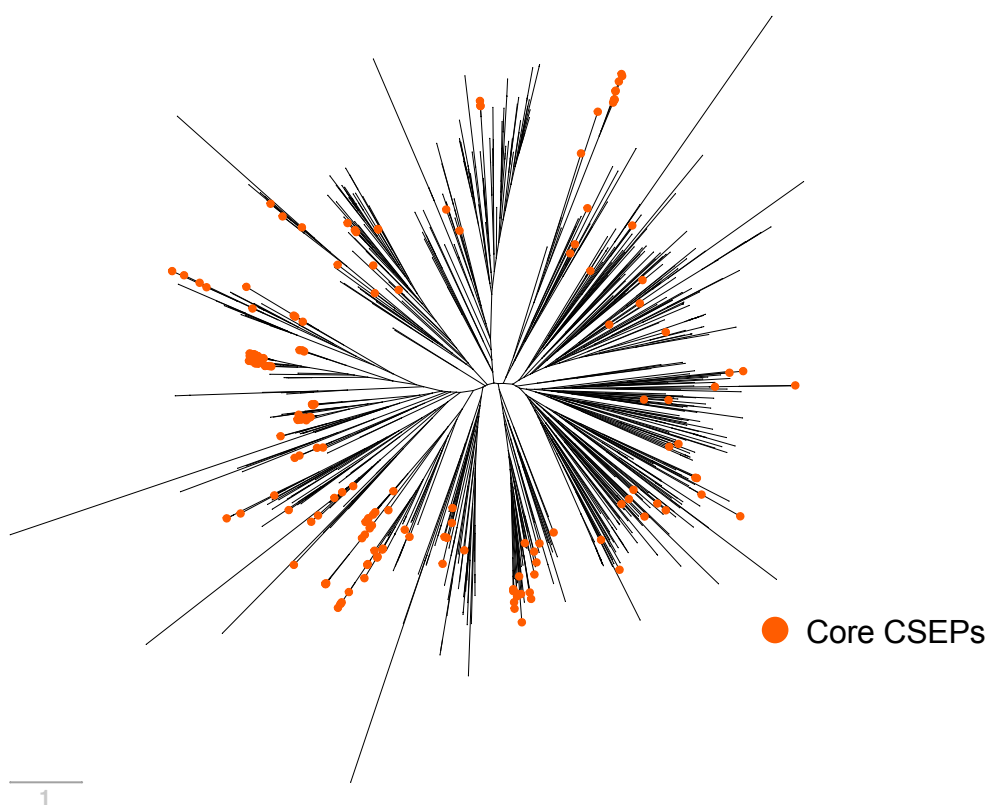

Supplement: Supplementary file 11 — Figure S10. Secretome orthology relations and core effectorome phylogeny. (A) Heatmap of SP orthologs found for the formae speciales genomes after ortholog clustering using OrthoFinder on the predicted proteomes of the isolates T1–20, S1459, LIB1609, DAC, 96224, LOL, AVE, POAE, DH14. Every column corresponds to one of the 805 Bgh DH14 predicted SPs, while color-coding depicts the number of orthologs in the corresponding orthogroup. Hierarchical clustering (Euclidean method) for the formae speciales and the SPs are given on the left and the top of the heatmap, respectively. (B) Maximum likelihood phylogeny tree of the 805 SPs. The tree was generated using IQ-TREE based on the mature peptide sequences of the Bgh DH14 SPs. Orange edge tips indicate the 190 core CSEPs which have orthologs in all formae speciales. The scale bar indicates the number of amino-acid substitutions per site. (PDF 2195 kb) [file 12864_2018_4750_MOESM11_ESM.pdf]

**A**

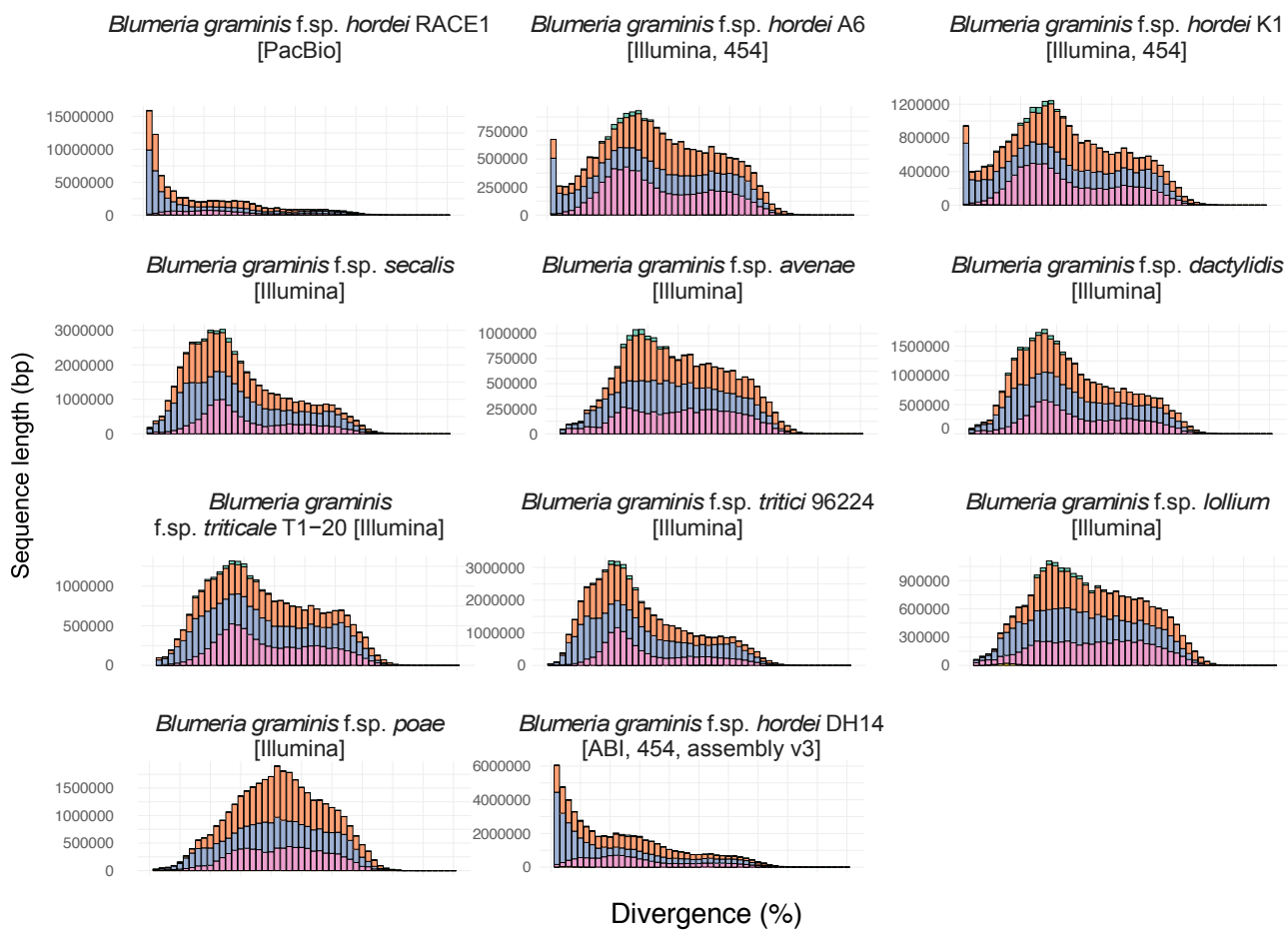

**B**

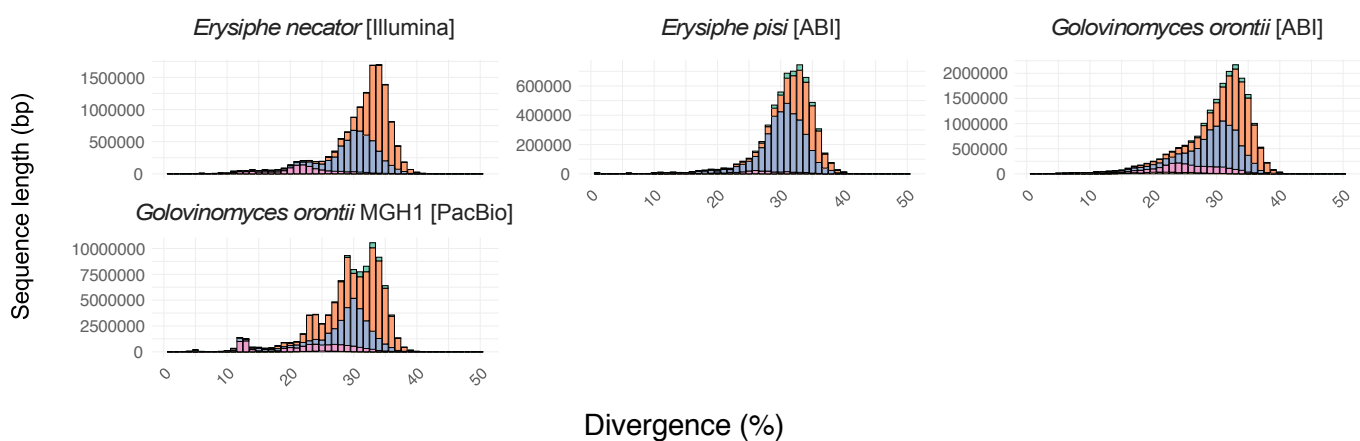

Type of TE

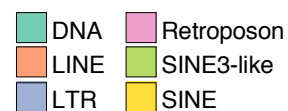

Supplement: Supplementary file 12 — Figure S11. Representatives of the genus Blumeria show less TE divergence than representatives of the genera Erishyphe and Golovinomyces. (A) The histograms indicate the frequency of a given sequence divergence for TE families of 10 B. graminis genomes. The genomes, which were assembled based on various sequencing platforms (PacBio or Illumina), were surveyed for their repeat content and repeat landscapes for each genome based on % nucleotide divergence to the consensus TE sequences were calculated out of the RepeatMasker output using Perl scripts. Sequence divergence (x-axis) is plotted against frequency (number of sequences; y-axis) for each of the genomes. (B) The histograms indicate the frequency of a given sequence divergence for TE families of 3 dicot-infecting powdery mildew species (Erysiphe pisi, E. necator and Golovinomyces orontii). The genomes, which were assembled based on various sequencing platforms (PacBio, ABI Solid or Illumina), were surveyed for their repeat content and repeat landscapes for each genome based on % nucleotide divergence to the consensus TE sequences were calculated out of the RepeatMasker output using Perl scripts. Sequence divergence (x-axis) is plotted against frequency (number of sequences; y-axis) for each of the genomes. (PDF 255 kb) [file 12864_2018_4750_MOESM12_ESM.pdf]
