# Supplementary material for: Signatures of host specialization and a recent transposable element burst in the dynamic one-speed genome of the fungal barley powdery mildew pathogen
Source: BMC Genomics. 2018 May 22;19:381. doi: 10.1186/s12864-018-4750-6 (PMC5964911; doi:10.1186/s12864-018-4750-6)
Supplement: Supplementary file 6 — Figure S5. Variation in the mating type locus in the Bgh isolates DH14 and RACE1. Organization of the genomic loci containing the mating type genes (MAT-1-1-1, MAT-1-1-3 and MAT-1-2-1) and some of its flanking genes. As DH14 and RACE1 are of opposite mating types, the structure of the mating type locus differs between the two isolates. The genomic locus in RACE1, which is of the MAT-1-1 mating type, was assembled completely, while the respective locus in DH14 (MAT-1-2 mating type) is distributed on two scaffolds. (PDF 1139 kb) [file 12864_2018_4750_MOESM6_ESM.pdf]

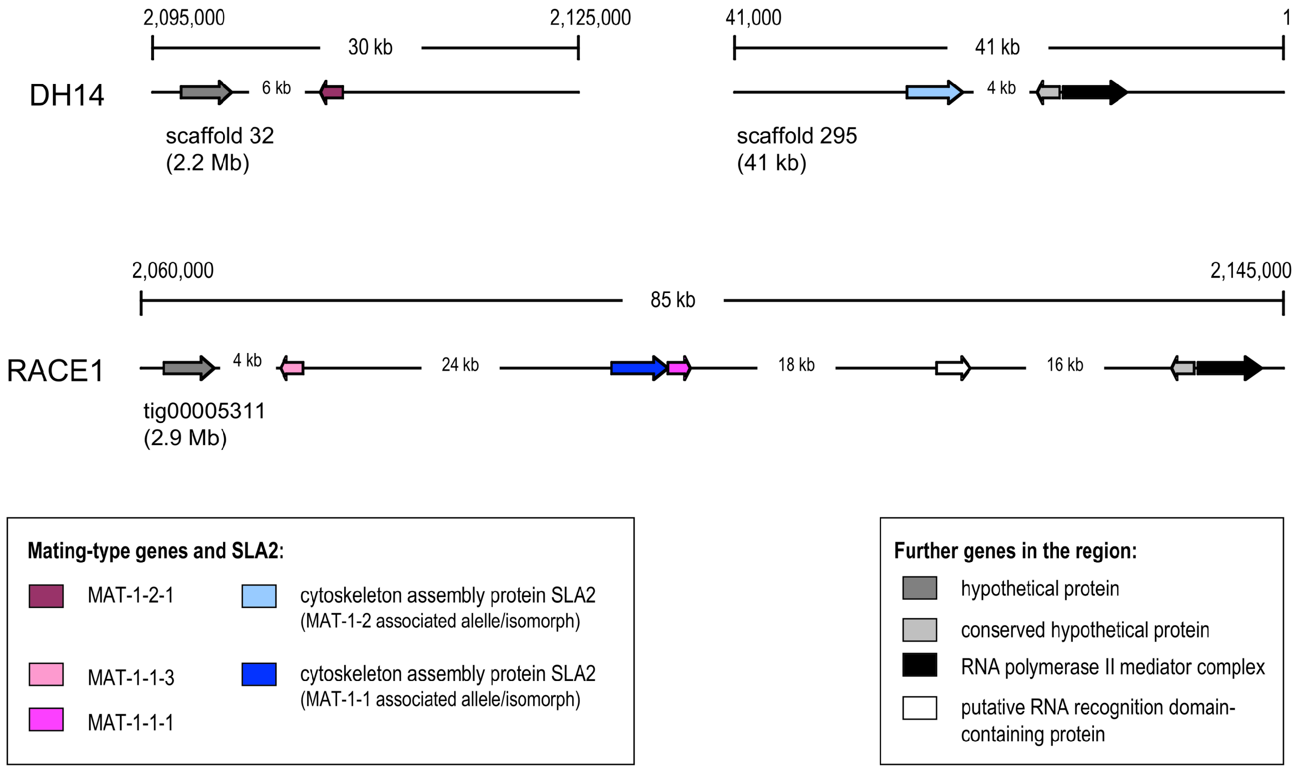
2,095,000
2,125,000
30 kb
DH14
6 kb
scaffold 32
(2.2 Mb)
41,000
1
41 kb
4 kb
scaffold 295
(41 kb)
2,060,000
2,145,000
85 kb
RACE1
4 kb
24 kb
18 kb
16 kb
tig00005311
(2.9 Mb)
Mating-type genes and SLA2:
MAT-1-2-1
cytoskeleton assembly protein SLA2
(MAT-1-2 associated alelle/isomorph)
MAT-1-1-3
cytoskeleton assembly protein SLA2
(MAT-1-1 associated allele/isomorph)
MAT-1-1-1
Further genes in the region:
hypothetical protein
conserved hypothetical protein
RNA polymerase II mediator complex
putative RNA recognition domain-
containing protein
