# Supplementary material for: Signatures of host specialization and a recent transposable element burst in the dynamic one-speed genome of the fungal barley powdery mildew pathogen
Source: BMC Genomics. 2018 May 22;19:381. doi: 10.1186/s12864-018-4750-6 (PMC5964911; doi:10.1186/s12864-018-4750-6)
Supplement: Supplementary file 2 — Figure S1. Comparative alignment of the Bgh DH14 and RACE1 genome assemblies with a Bgh genetic map. The distribution and ordering of 80 single copy EST markers across 30 linkage groups of a previously published genetic map [22] is visualized in relation to the corresponding genomic locations of these markers in the DH14/RACE1 assemblies. Each box represents a specific genomic contig or linkage group (LG), respectively, and the numbers inside the boxes specify the marker positions on the corresponding contig (in bp) or linkage group (in cM). The corresponding marker identifiers are given next to the boxes. Dashed connector lines represent markers for which the genomic location and genetic map are consistent. Discrepancies between assembly and genetic map are indicated by solid connectors, with black lines representing markers whose location is consistent between assemblies but different from the genetic map, and colored lines representing markers with differences to the genetic map that are specific to either DH14 (dark pink) or RACE1 (blue). (PDF 4159 kb) [file 12864_2018_4750_MOESM2_ESM.pdf]

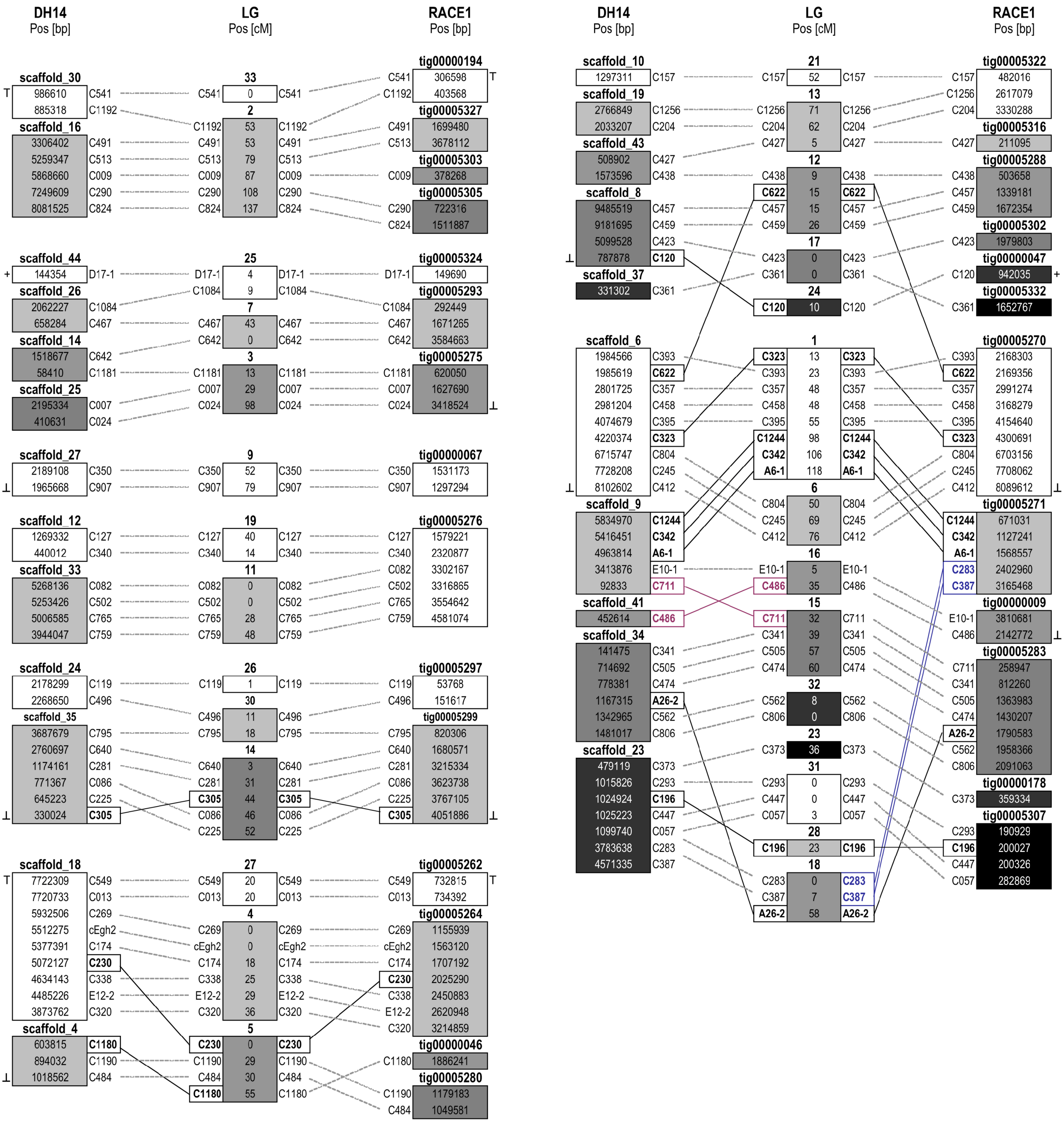
DH14
Pos [bp]
LG
Pos [cM]
RACE1
Pos [bp]
scaffold_30
986610 C541
885318 C1192
scaffold_16
3306402 C491
5259347 C513
5868660 C009
7249609 C290
8081525 C824
33
C541 0 C541
2
C1192 53 C1192
C491 53 C491
C513 79 C513
C009 87 C009
C290 108 C290
C824 137 C824
tig00000194
C541 306598
C1192 403568
tig00005327
C491 1699480
C513 3678112
tig00005303
C009 378268
tig00005305
C290 722316
C824 1511887
scaffold_44
144354 D17-1
scaffold_26
2062227 C1084
658284 C467
scaffold_14
1518677 C642
58410 C1181
scaffold_25
2195334 C007
410631 C024
25
D17-1 4 D17-1
C1084 9 C1084
7
C467 43 C467
C642 0 C642
3
C1181 13 C1181
C007 29 C007
C024 98 C024
tig00005324
D17-1 149690
tig00005293
C1084 292449
C467 1671265
C642 3584663
tig00005275
C1181 620050
C007 1627690
C024 3418524
scaffold_27
2189108 C350
1965668 C907
9
C350 52 C350
C907 79 C907
tig00000067
C350 1531173
C907 1297294
scaffold_12
1269332 C127
440012 C340
scaffold_33
5268136 C082
5253426 C502
5006585 C765
3944047 C759
19
C127 40 C127
C340 14 C340
11
C082 0 C082
C502 0 C502
C765 28 C765
C759 48 C759
tig00005276
C127 1579221
C340 2320877
C082 3302167
C502 3316865
C765 3554642
C759 4581074
scaffold_24
2178299 C119
2268650 C496
scaffold_35
3687679 C795
2760697 C640
1174161 C281
771367 C086
645223 C225
330024 C305
26
C119 1 C119
30
C496 11 C496
C795 18 C795
14
C640 3 C640
C281 31 C281
C305 44 C305
C086 46 C086
C225 52 C225
tig00005297
C119 53768
C496 151617
tig00005299
C795 820306
C640 1680571
C281 3215334
C086 3623738
C225 3767105
C305 4051886
scaffold_18
7722309 C549
7720733 C013
5932506 C269
5512275 cEgh2
5377391 C174
5072127 C230
4634143 C338
4485226 E12-2
3873762 C320
scaffold_4
603815 C1180
894032 C1190
1018562 C484
27
C549 20 C549
C013 20 C013
4
C269 0 C269
cEgh2 0 cEgh2
C174 18 C174
C338 25 C338
E12-2 29 E12-2
C320 36 C320
5
C230 0 C230
C1190 29 C1190
C484 30 C484
C1180 55 C1180
tig00005262
C549 732815
C013 734392
tig00005264
C269 1155939
cEgh2 1563120
C174 1707192
C230 2025290
C338 2450883
E12-2 2620948
C320 3214859
tig00000046
C1180 1886241
tig00005280
C1190 1179183
C484 1049581
scaffold_10
1297311 C157
scaffold_19
2766849 C1256
2033207 C204
scaffold_43
508902 C427
1573596 C438
scaffold_8
9485519 C457
9181695 C459
5099528 C423
787878 C120
scaffold_37
331302 C361
21
C157 52 C157
13
C1256 71 C1256
C204 62 C204
C427 5 C427
12
C438 9 C438
C622 15 C622
C457 15 C457
C459 26 C459
17
C423 0 C423
C361 0 C361
24
C120 10 C120
tig00005322
C157 482016
C1256 2617079
C204 3330288
tig00005316
C427 211095
tig00005288
C438 503658
C457 1339181
C459 1672354
tig00005302
C423 1979803
tig00000047
C120 942035
tig00005332
C361 1652767
scaffold_6
1984566 C393
1985619 C622
2801725 C357
2981204 C458
4074679 C395
4220374 C323
6715747 C804
7728208 C245
8102602 C412
scaffold_9
5834970 C1244
5416451 C342
4963814 A6-1
3413876 E10-1
92833 C711
scaffold_41
452614 C486
scaffold_34
141475 C341
714692 C505
778381 C474
1167315 A26-2
1342965 C562
1481017 C806
scaffold_23
479119 C373
1015826 C293
1024924 C196
1025223 C447
1099740 C057
3783638 C283
4571335 C387
1
C323 13 C323
C393 23 C393
C357 48 C357
C458 48 C458
C395 55 C395
C1244 98 C1244
C342 106 C342
A6-1 118 A6-1
6
C804 50 C804
C245 69 C245
C412 76 C412
16
E10-1 5 E10-1
C486 35 C486
15
C711 32 C711
C341 39 C341
C505 57 C505
C474 60 C474
32
C562 8 C562
C806 0 C806
23
C373 36 C373
31
C293 0 C293
C447 0 C447
C057 3 C057
28
C196 23 C196
18
C283 0 C283
C387 7 C387
A26-2 58 A26-2
tig00005270
C393 2168303
C622 2169356
C357 2991274
C458 3168279
C395 4154640
C323 4300691
C804 6703156
C245 7708062
C412 8089612
tig00005271
C1244 671031
C342 1127241
A6-1 1568557
C283 2402960
C387 3165468
tig00000009
E10-1 3810681
C486 2142772
tig00005283
C711 258947
C341 812260
C505 1363983
C474 1430207
A26-2 1790583
C562 1958366
C806 2091063
tig00000178
C373 359334
tig00005307
C293 190929
C196 200027
C447 200326
C057 282869
